# KYLO-0603, a novel liver-targeting, thyroid hormone receptor-β agonist for the inhibition of MASH progression

**DOI:** 10.1371/journal.pone.0331768

**Published:** 2025-09-15

**Authors:** Xueqin lu, Shengjun Wang, Yanchun Du, Bixian Xie, Qingyan Chen, Jinzhen Lin, Bailing Chen, Kunyuan Cui

**Affiliations:** 1 Kylonova (Xiamen) biopharma Co., Ltd, Xiamen, China; 2 Hygieia pharmaceuticals Co., Ltd, Hangzhou, China; Zhejiang University of Technology, CHINA

## Abstract

Metabolic dysfunction–associated steatohepatitis (MASH) is a progressive liver disease associated with liver-related complications and death. Kylo-0603 is a novel agonist for the thyroid hormone receptor β (THR-β) that has been developed by merging the structures of three acetylgalactosamine (GalNAc)-modified ASPGR ligands with a triiodothyronine (T3) analog. This unique design enables both THR-β activation and targeted delivery to hepatocytes, which significantly reduces the risk of adverse effects related to increased systemic thyroid hormone activity. Additionally, it effectively lowers serum cholesterol levels by as much as 69.2% and low-density lipoprotein cholesterol (LDL-C) levels by up to 88.2% in the MASH mouse model. Meanwhile, Kylo-0603 demonstrated a marked improvement in histological parameters, decreasing steatosis by up to 1.3 points (P < 0.001), inflammation by 1.8 points (P < 0.0001), and ballooning by 0.8 points (P < 0.01). The non-alcoholic steatohepatitis (NASH) activity score (NAS) demonstrated a significant reduction of up to 3.7 points (P < 0.0001), while the fibrosis score decreased by 0.6 points (P < 0.05). These findings indicate that Kylo-0603 effectively ameliorates hepatic MASH pathology and attenuates fibrosis progression. In summary, Kylo-0603-a highly tissue- and target-selective, low-toxicity THR-β agonist—exhibits substantial therapeutic potential for managing MASH and represents a promising novel treatment option for affected patients.

## Introduction

The liver is an essential organ closely related to lipid metabolism and is responsible for both the regulation of lipid homeostasis and energy utilization [1,2]. Metabolic dysfunction-associated steatotic liver disease (MASLD), formerly known as nonalcoholic fatty liver disease (NASLD), can be diagnosed in adults with hepatic steatosis detected by imaging techniques, blood biomarkers, or liver histology, when overweight or obese, or in the presence of T2DM or at least two metabolic risk abnormalities [3]. Currently, MASLD is the most common liver disease worldwide, affecting approximately 30% of the adult population. Regional epidemiological data indicate that the highest prevalence rates are observed in the Middle East (32%) and South America (30%), and the lowest is found in Africa (13%). It is worth noting that prevalence rates are even higher in specific subpopulations such as severely obese patients (90%) and patients with type 2 diabetes (76%). Although liver steatosis is usually benign, it can progress to MASH, previously termed nonalcoholic steatohepatitis (NASH) [4,5]. MASH is an important sign of disease progression, and, if not controlled, can progress to cirrhosis and even liver cancer [6–10]. In contrast to simple steatosis where the primary histological feature is lipid accumulation in liver cells, MASH is characterized by additional liver inflammation with or without fibrosis. Chronic steatosis drives the disease progression toward MASH, which is characterized by inflammation and ballooning or hepatocyte damage [11–13]. In recent decades, many researchers and pharmaceutical companies have made great efforts to develop drugs for MASLD and MASH in an attempt to inhibit the MASH process. Despite extensive research into the pathogenesis and drug development of MASH, only one drug that targets the treatment of MASH was approved by the FDA (U.S. Food and Drug Administration) in 2024 [7,14–16].

Thyroid hormones (THs) are considered to be important signaling molecules for maintaining normal body metabolism and play critical roles in differentiation, growth, and metabolism [17,18]. They exert their physiological effects by binding to specific nuclear receptors, THR-α and THR-β, which are differentially expressed and control tissue/cell-specific thyroxine activity [19–20]. In contrast to the THR-α receptor, which is predominantly expressed in the heart, the THR-β isoform is primarily expressed in the liver and exerts significant effects on lipid metabolism [21–23]. Specifically, THR-β has been demonstrated to reduce LDL-C levels, decrease generalized obesity and body weight, and is capable of reducing lipid content by increasing the rate of lipid metabolism in the liver [24,25]. Moreover, Perra et al. demonstrated that THR agonists have the potential to inhibit or reverse hepatic steatosis [26]. This significant discovery highlights the remarkable therapeutic potential of THR agonists in the treatment of MASLD. Although several THR-β agonists, including Resmetirom, which has which has received marketing approval from the FDA, none of these compounds are liver-specific targeting [20,27,28].

The hepatic asialoglycoprotein receptor (ASGPR) is a highly abundant, rapidly internalized receptor with many cellular lectins selectively distributed on the surface of hepatocytes [29–31]. Modification of drug formulations with ASGPR ligands can significantly improve drug selectivity and permeability to hepatocytes [32–34]. ASGPR promotes the uptake and clearance of circulating glycoproteins with exposed terminal galactose and n-acetylgalactosamine, amino sugar derivatives of galactose and glycans, via lattice protein-mediated endocytosis [35–37]. Thus, a consensus has been reached that triantennary GalNAc with a mutual distance of ∼20 Å exhibits the highest affinity with ASGPR, which has been widely used for liver-targeted delivery of various payloads [38,39].

**Fig 1:**
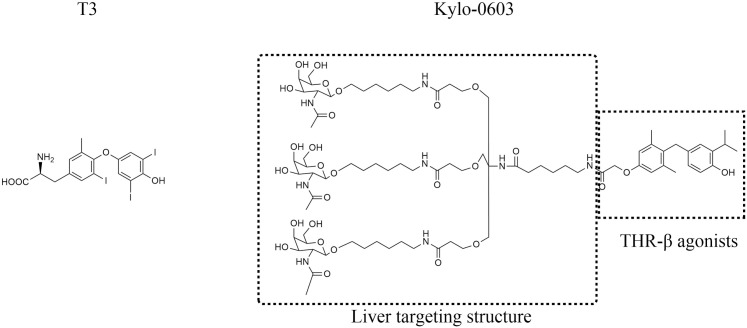
The structures of T3 and Kylo-0603.

The present study was undertaken to mitigate the adverse effects of therapeutic agents for MASLD or MASH and to enhance the accumulation of THR-β agonists in the liver while maintaining the regulatory role of thyroxine on intra-hepatic lipid metabolism. To this end, a series of innovative compounds incorporating the liver-targeting ligand Gal-NAc with thyroxine receptor agonists were designed and synthesized (see our patent US11690818B2). Following a period of careful screening, Kylo-0603 was identified as the optimal candidate (Fig 1). The data from the study demonstrated that, following an eight-week treatment period, Kylo-0603 resulted in a significant improvement in MASH and/or fibrosis in the MASH mouse model (F2 ~ 3) induced with a high-fat diet (HFD) in conjunction with carbon tetrachloride (CCl_4_) induction while simultaneously reducing LDL-C and body weight. This provides an additional therapeutic advantage for patients with MASH associated with hyperlipidemia or obesity.

## Materials and methods

### Chemistry

All reactions were run under an inert atmosphere (Ar) with flame-dried glassware using standard techniques for manipulating air-sensitive compounds. All the solvents were dried and purified before use by standard procedures. Commercial reagents were used as supplied or purified by standard techniques where necessary. Column chromatography was performed using 200–300 mesh silica with the proper solvent system according to TLC analysis and UV light to visualize the reaction components. Unless otherwise noted, nuclear magnetic resonance (NMR) spectra were recorded on a 400 MHz spectrometer. NMR data were reported as follows: chemical shift, multiplicity (s = singlet, d = doublet, dd = doublet-doublet, t = triplet, q = quartet, qt = quartet of triplets, m = multiplet), coupling constant in Hz and integration. Chemical shifts of ^1^H NMR spectra were recorded in parts per million (ppm) on the δ scale from an internal standard of residual CDCl_3_ (7.26 ppm), and CD_3_CN (1.94 ppm). Chemical shifts for ^13^C NMR spectra were recorded in parts per million from tetramethylsilane using the central peak of CDCl_3_ (77.16 ppm), and CD_3_CN (1.32, 118.26 ppm) as the internal standard. HR-MS data were obtained using ESI ionization with 100,000 (FWHM) maximum resolution.

### THR-β selectivity assessment of Kylo-0603

In conducting experiments to ascertain the agonist effects of compounds on human THR-α or THR-β, the following equipment and reagents were utilized: The experimental apparatus comprised an Envision instrument (provided by PerkinElmer, model Envision 2014) and an Echo 500 instrument from Labcyte, USA. The experimental reagents used were the LanthaScreen™ TR-FRET THRα/THRβ Coactivator Detection Kit (model PV4687) from Thermo Fisher Scientific. The procedure was as follows: initially, gradient dilutions of the drugs Kylo-0603 and T3 were conducted on the Echo 500 instrument. The dilution was conducted via a 10-point, 3-fold serial dilution, with the initial concentration of Kylo-0603 ranging from 400 μM to 40 μM and that of T3 ranging from 40 nM to 2 nM. Subsequently, 100 nL of the diluted compounds were transferred to a 384-well experimental plate (384 Optiplate), with one compound well designated for each concentration. The experimental procedure was conducted by the instructions provided in the LanthaScreen™ TR-FRET TRα/TRβ Coactivator Assay Kit. To ensure the accuracy and reliability of the data, each experiment was repeated three times. EC50 values were determined by using GraphPad Prism 8.5 (the Dose-response-Stimulation-log[effect] vs. response--Variable slope). Finally, to assess the selectivity of the compounds, we calculated the selectivity multiplicity. Fold of selectivity was calculated by (compound EC50 on THR-α/T3 EC50 on THR-α)/ (compound EC50 on THR-β/T3 EC50 on THR-β)

### Animal study

This experimental protocol and any changes to the experimental protocol involving the use and care of animals were reviewed and approved by the Institutional Animal Care and Utilization Committee (IACUC) committee before implementation. During the experiments, animal welfare and experimental procedures followed AAALAC animal welfare requirements. The animals were maintained on a 12/12 h light/dark cycle with free access to water and food. All animals come from GemPharmatech Co., Ltd. The health status of all experimental animals was monitored daily and there were no abnormalities observed during the study. Following the IACUC-approved animal care standard operating procedure (SOP), any animal abnormalities would be immediately reported to the responsible study director and veterinarian. Depending on the conditions, necessary steps, such as bandaging, transfer to single-animal housing, dosing pause, temperature adjustments, or nutrition supplementation, would be taken to minimize animal suffering and distress. Anesthesia with isoflurane and euthanasia by CO_2_ inhalation were used in this study following the IACUC-approved SOP.

### Experimental observation and data collection of HFD-induced obese mice

Experiments were performed on 112 (n = 16/group) male C57BL/6J mice (5 weeks). After 3 days of acclimatization, the animals were randomly divided according to body weight into a control group (Chow Diet) and a HFD group (HFD, 60% calories as fat, Cat# D12492, Research Diet, Jiangsu Xietong Pharmaceutical Bioengineering Co., Ltd.). The mice were fed with the corresponding diets for 16 weeks (with bedding shavings and a high-fat diet changed twice a week), during which the bedding shavings and high-fat diet (HFD) were refreshed twice weekly (see S1 Fig). Body weights were recorded on a weekly basis throughout this period. In the meantime, Kylo-0603 (0.1 mg/kg to 10 mg/kg) or vehicle was orally administered to HFD-induced obese mice once daily (S1 Table and S2 Table). A separate cohort of mice, fed a standard chow diet, served as normal controls and received daily oral gavages of the vehicle. At the end of the experiment, the mice were fasted for 6 hours and then sacrificed. The animals were euthanized by CO_2_ inhalation and blood were collected along with liver and heart tissues. Collected blood samples were centrifuged to obtain the serum for the detection of cholesterol, triglyceride, and low-density lipoprotein cholesterol. The heart and liver tissues were weighed and photographed. The livers and hearts of the mice were rapidly removed and washed with cold saline. Half of the left lobe of liver was fixed in 4% neutral paraformaldehyde, and the remaining part of livers and hearts were snap-frozen and stored at −80°C for further analysis. The concentrations of serum lipids and liver enzymes from orbital or cardiac blood were determined by measuring triglycerides (TG), cholesterol (Chol), Low-density lipoprotein cholesterol (LDL-C), alanine transaminase (ALT), and aspartate transaminase (AST) levels using a Hitachi 7020 automated hematology laboratory and a Wako kit.

### Experimental observation and data collection of MASH mouse model (F2 ~ 3) induced with a high-fat diet (HFD) in conjunction with carbon tetrachloride (CCl_4_) induction

A total of 80 5-week-old C57BL/6J male mice were randomly assigned to two groups based on their body weight: a control group (n = 10) and a high-fat diet group (HFD, D12492, n = 70). During the eight-week feeding period, the mice in the two groups were provided with the corresponding diets, including shavings bedding, and their weights were recorded once a week. The change of the weight of each group of mice at a given time were recorded. The 60 mice exhibiting the highest body weights in the 60% high-fat diet group were selected and subsequently andomized into six groups, each consisting of 10 mice, each based on body weight to ensure homogeneity within groups. The control diet group was provided with a control diet supplemented with the solvent vehicle, while the HFD control group was given a high-fat diet with the same supplement. The remaining four groups were provided a high-fat diet containing the solvent and administered a range of doses of Kylo-0603, namely, 0.1 mg/kg, 0.3 mg/kg, 1 mg/kg, 3 mg/kg, and 10 mg/kg. The HFD control group was administered intraperitoneal injections of 0.2 ml/kg CCl_4_ twice a week for 8 weeks, during which time the mice were fed the same diets as before. The mice were weighed weekly, and the dosage for each week was determined based on their body weight at the commencement of that week. After a period of fasting of between 5 and 6 hours on the day following the final administration of the drug, the mice in each group were euthanized, whole blood was collected from either the orbits or the heart, and the serum was separated and stored at −80°C. The heart and liver tissues of the mice in each group were weighed and photographed. The heart tissues were then quickly frozen in liquid nitrogen and stored in a refrigerator at −80°C, and the middle lobe of the liver was divided into four parts. Two of these samples were quickly frozen in liquid nitrogen and stored at −80°C (for RNA sequencing). The remaining two portions were fixed in a 4% Paraformaldehyde solution and subsequently dehydrated for paraffin embedding and OCT embedding. The serum concentrations of TG, Chol, LDL-C, ALT, and AST levels were determined with a Hitachi 7020 automated hematology laboratory and a Wako kit. Meanwhile the sera were separated from the blood collected via cardiac puncture in each group with Cloud-Clone products and tested according to the instructions provided with the thyroid stimulating hormone (TSH, also known as thyrotropin), triiodothyronine (T3), thyroxine (T4), free T4 (fT4) and free T3 (fT3) enzyme immunoassay kits. For the quantification of hepatic lipid content, liver tissues were meticulously homogenized in isopropanol (1:9 tissue to isopropanol by mass/volume) on ice. The homogenate was then incubated overnight at 4°C and subsequently centrifuged to remove the supernatant. The resulting solution was analyzed for TG and Chol using a Hitachi 7020 automated hematochemistry system.

### Hematoxylin and eosin (H&E) Staining

The dewaxed tissue slides were incubated in the alcohol-free hematoxylin staining solution for a period of 3–5 minutes, followed by thorough rinsing with running tap water. The slides were then subjected to differentiated and bluing for several seconds, after which they underwent sequential dehydration through a series of ethanol solutions with increasing concentrations (50%, 70%, 85%, and 95%). They were then subjected to eosin staining for a period of 5–10 minutes. The slides were then dehydrated sequentially with 95%, 100%, and 100% ethanol before being cleared with xylene. Finally, the slides were sealed with neutral gum, dried, and imaged.

### Oil Red Staining

The frozen sections were immersed in 60% isopropyl alcohol for 3–5 minutes, followed by immersion in Oil Red staining solution for 3–5 minutes. The samples were then washed with pure water. Following a wash with pure water, the nuclei were restained with hematoxylin, after which the sections were sealed with 50% glycerol, dried and photographed for imaging.

### Sirius Red Staining

The dewaxed slides were rinsed in running water, stained with Sirius Red for 8–10 minutes, washed slightly in anhydrous ethanol, dehydrated, made transparent by gradient ethanol and xylene, and finally sealed with neutral gum, dried and photographed for imaging.

### Statistical analysis

The results of the experiments, including the body weights of the animals in each group, are expressed as the means ± standard deviations (means ± SD). Statistical analysis was performed in GraphPad Prism 8.0 and Microsoft Excel. The analysis varied for different datasets and details on the procedures are reported in the figure legends.

### SHG/TPEF microscopy and imaging procedure

Scanning was conducted on a Genesis® machine, a stain-free TPE/SHG microscopic imaging device manufactured in-house by HistoIndex®, Singapore. The region of interest (ROI) is systematically partitioned into uniformly sized, termed “blocks,” each measuring of 200 μm × 200 μm. These blocks were sequentially imaged individually and in sequence in raster scan mode utilizing a 20x objective lens. Each block has a resolution of 512 × 512 pixels, or 0.4 μm per pixel. Software developed by HistoIndex and Chipmap for image analysis identifies the different lobular regions, the central vein, the confluent region, and the perisinus region. Fibrosis and steatosis in these regions were subsequently measured and quantified in the SHG and TPE images.

### PK studies

The selected compounds were individually administered orally to ICR rats (male, n = 3 per time point) separately. Blood samples were collected before and at 0.25, 0.5, 1, 2, 4, 8, and 24 h after oral administration and then centrifuged at 4500 rpm for 10 min at 4°C to obtain the serum. Liver samples were collected before dosing and at 1, 4, 8, and 24 h after oral administration. The concentrations of the compounds in the serum or liver samples were quantified using liquid chromatography/tandem mass spectrometry (LC − MS/MS).

### Gene expression analysis

Transcriptome sequencing was performed on the Illumina sequencing platform, and the library sequencing and biotechnological analysis were performed by Beijing Novozymes Technology Co.

## Results

### Design and synthesis of Kylo-0603

**Fig 2:**
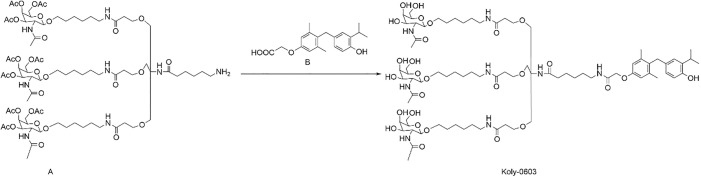
Synthesis process of Kylo-0603.

This study is focused on the design and synthesis of an novel liver-targeting compound, Kylo-0603, which integrates the highly liver-targeting properties of GalNAc with the biological activity of a thyroid hormone receptor agonist. Specifically, Kylo-0603 was prepared by chemically coupling Compound B with Compound A (a GalNAc derivative, the synthesis step of which in details is shown in the supporting document (see S2–S7 Fig). The simplified synthesis of Kylo-0603 is shown in Fig 2. The specific experimental procedure was to sequentially add DMF (3.0 mL), Compound B (15 mg), TBTU (8.47 mg), and DIPEA (20.2 mg) to the reaction vessel and react for 6 hours. Compound A (47 mg) was then rapidly added and stirred for 2 hours at room temperature. The reaction was detected by high-performance liquid chromatography (HPLC), and the reaction was completed and terminated. The reaction mixture was prepared with a 1.0 mol/L ammonia solution in an ice bath to ensure that the pH of the reaction mixture was 8−10. The ice bath was removed, and the mixture was stirred at room temperature for half an hour while HPLC detection was performed to monitor the completeness of reaction. At the end of the reaction, the pH was adjusted to 7.0 with glacial acetic acid, and the mixture was then concentrated. The concentrated residue was dissolved in 35% acetonitrile/water, filtered and lyophilized to yield 29.47 mg of the target compound. ^1^H NMR (400 MHz, DMSO-d_6_) δ 8.97 (s, 1H), 8.01 (d, J = 5.2 Hz, 1H), 7.82 (t, J = 5.2 Hz, 3H), 7.65 (d, J = 8.8 Hz, 3H), 7.00 (s, 1H), 6.82 (s, 1H), 6.65 (s, 2H), 6.61 (d, J = 8.0 Hz, 1H), 6.45 (d, J = 7.6 Hz, 1H), 4.61 (t, J = 5.2 Hz, 3H), 4.57 (d, J = 4.8 Hz, 3H), 4.48 (d, J = 2.8 Hz, 3H), 4.40 (s, 2H), 4.24 (d, J = 8.0 Hz, 3H), 3.79 (s, 2H), 3.73 (m, 3H), 3.69 (m, 3H), 3.53 (m, 24H), 3.34 (m, 6H), 3.12 (m, 3H), 3.03(d, J = 5.2 Hz, 6H), 2.29 (t, J = 5.6 Hz, 6H), 2.15 (s, 6H), 2.07 (t, J = 7.2 Hz, 3H), 1.82 (s, 9H), 1.24–1.43 (m, 30H), 1.09 (d, J = 6.8 Hz, 6H). ^13^C NMR (100 MHz, DMSO-d6)) 172.98, 170.57, 170.09, 168.23, 155.91, 152.70, 138.12, 134.35, 130.92, 130.28, 125.88,125.35, 115.28, 114.52, 101.84, 75.65, 71.98, 68.79, 68.54, 68.05, 67.83, 67.37, 60.99, 59.98, 52.64, 38.99, 38.69, 36.46, 36.34, 33.58, 29.63, 29.53, 29.36, 26.91, 26.69, 26.40, 25.66, 25.50, 23.47, 22.93, 20.51; HR-MS (ESI) m/z calcd for C_81_H_134_N_8_O_28_ [M + H] ^+^: 1667.9380, found: 1667.9382.

### Kylo-0603 drug stability determination and in vitro receptor binding assay

We evaluated the metabolic stability of Kylo-0603 in the plasma of CD-1 mice, Sprague–Dawley (SD) rats, beagle dogs, cynomolgus monkeys, and humans. The plasma from the five species was mixed with Kylo-0603 (2 μM) and incubated at 37°C in a thermostatic water tank. The plasma concentrations of Kylo-0603 were monitored via LC-MS/MS at 0, 10, 30, 60, and 120 min. The half-lives (T_1/2_) of Kylo-0603 in the plasma of CD-1 mice, SD rats, beagle dogs, cynomolgus monkeys and humans were determined to be > 372.7, > 372.7, 355.8, > 372.7 and >372.7 min, respectively. Kylo-0603 was stable in the plasma of CD-1 mice, SD rats, cynomolgus monkeys and humans and unstable in beagle dog plasma.

### THR-β selectivity assessment of Kylo-0603

**Fig 3:**
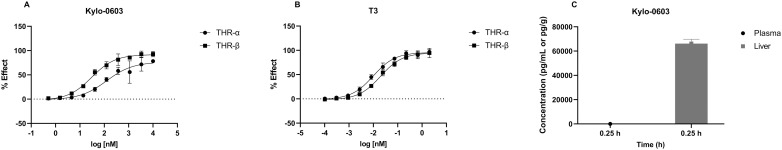
Kylo-0603’s EC50 & tissue levels. EC50 ratios of Kylo-0603 for the THR-α and THR-β receptors **(A)**. The EC50 ratios of T3 to THR-α and THR-β receptors **(B)**. Plasma and Liver Concentrations of Kylo-0603 after PO Dosing in Rats **(C).**

Kylo-0603 is a new compound based on a structural modification of the thyroid hormone T3 that incorporates GalNAc and a T3-like thyroid hormone structure, a design that gives Kylo-0603 the dual properties of a liver-targeting and THR-β receptor agonist. To assess the binding selectivity of Kylo-0603 for THR-α and THR-β receptors and compare it with that of T3, THR-α and THR-β receptor coactivator experiments were performed. The experimental results revealed that Kylo-0603 bound THR-α and THR-β with EC50 values of 124.1 ± 13.92 nM and 31.07 ± 4.42 nM, respectively, whereas T3 bound both receptors with EC50 values of 0.01 ± 0.002 nM and 0.02 ± 0.001 nM, respectively. After normalization for the relative selectivity of T3 binding to the receptors, the results revealed that the relative selectivity of Kylo-0603 for THR-β was 8.2-fold greater than that for THR-α (Fig 3A). Although Kylo-0603 demonstrated a relatively lower binding affinity for THRα compared to THR-β when compared with the established compound GC-1, it exhibited superior hepatic targeting efficacy relative to prior THRβ agonists. To evaluate the pharmacokinetic properties of the novel compound Kylo-0603, a single dose of 7.03 mg/kg was administered to fasted male Sprague‒Dawley (SD) rats. The plasma and hepatic drug concentrations were accurately analyzed using high-performance liquid chromatography-tandem mass spectrometry (HPLC-MS/MS). The results revealed a low plasma exposure level of Kylo-0603 (12.8 pg/ml at 0.25 h). In contrast, a notable accumulation of the drug was observed in the liver, where the concentration reached an exceptionally high level of 66167 pg/g at 0.25 hours post-dosing. This finding demonstrates that the drug has specific targeting properties in the liver (Fig 3C).

### Effects of Kylo-0603 on body weight and fat content in HFD-induced obese mice

**Fig 4:**
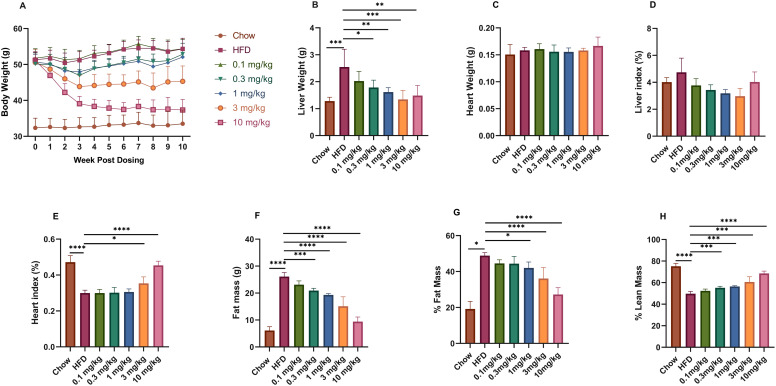
Effects of Kylo-0603 on body weight, organ weight, and body fat content. Male 5-week-old C57BL/6J mice were fed either a normal diet or a high-fat diet (HFD; 60 kcal% fat) and maintained for 16 weeks. The animals were subsequently administered a single daily oral dose of Kylo-0603 for 10 weeks in conjunction with HFD feeding. Body weights of the mice after drug intervention **(A)**. Liver and heart weights of the mice after 10 weeks of drug intervention **(B and C)**. Relative fat mass% (E) and lean mass% (F) after 8 weeks of Kylo-0603 treatment. Body fat and lean mass percentages were calculated by dividing body fat volume and body lean mass volume by total volume, respectively. Data are shown as the mean±SD, n = 5; * P < 0.05, **P < 0.01, ***P < 0.001, and ****P < 0.0001 vs the high-diet control group by one-way ANOVA with Dunnett’s post hoc test.

Compared with those in the standard diet control group (chow), the high-fat diet (HFD)-induced obesity model mice in this study presented significant increases in body weight, liver weight and body fat percentage (S8 Fig). During the experimental period, there was no significant difference in food intake between the control and experimental groups. Following daily administration of a single dose of Kylo-0603 for 10 weeks in conjunction with HFD feeding, the body weights of the mice in the Kylo-0603 groups at doses of 0.1, 0.3, 1, 3, and 10 mg/kg were reduced by 0.8%, 4.8%, 3.8%, 13.1%, and 15.8%, respectively, compared to those of the mice in the HFD control group (Fig 4A). The liver weight and body weight of the mice significantly decreased in all the dose groups, but the effect of various doses of Kylo-0603 on the heart weight of the mice was not significant (Fig 4B and Fig 4C). An interesting dose-response relationship was observed with the drug intervention: the 10 mg/kg dose significantly reduced the body weight of the mice, resulting in a significant increase in the heart weight/body weight ratio at this dose (Fig 4E). However, the liver weight decreased with increasing concentrations of the drug administered, so the liver weight/body weight ratio essentially remained unchanged at the low dose and increased only at the highest dose of 10 mg/kg (Fig 4D). Body length and bone density were not affected by Kylo-0603 (S9 Fig).

Further detailed posttreatment analysis revealed that Kylo-0603, which was administered at five different doses, significantly reduced the fat content in a dose-dependent manner (Fig 4F). The specific reductions were 8.96%, 9.16%, 14.07%, 26.15%, and, most notably, 44.35%, demonstrating its potent fat-reducing effects (Fig 4G). In addition, Kylo-0603 significantly increased lean mass in mice while reducing fat content. Following an eight-week administration period, there was a notable increase in the percentage of lean mass by 5.32%, 10.95%, 13.52%, 21.94%, and 37.91%, respectively, in comparison to the HFD control group (as illustrated in Fig 4H). Given that lean mass is essentially constant (S9B Fig), the observed increase in lean percentage content was not attributable to an increase in lean mass; rather, it was due to a decrease in fat content. These characteristics are not observed with CG-1 administration [40].

### Lipid-lowering effects in HFD-induced obese mice

After 10 weeks of Kylo-0603 drug intervention, compared with those in the HFD group, the levels of Chol and LDL-C in the blood of the mice in all the drug-treated groups were significantly decreased in a dose-dependent manner. Eight mice were randomly selected from each group for lipid parameters determination (including Chol and LDL-C) at week 2 and the end of treatment (week 10). The results of the study demonstrated that Kylo-0603 was effective at reducing plasma cholesterol and LDL-C levels in mice at all the tested doses and that this effect was significantly dose-dependent.

**Fig 5:**
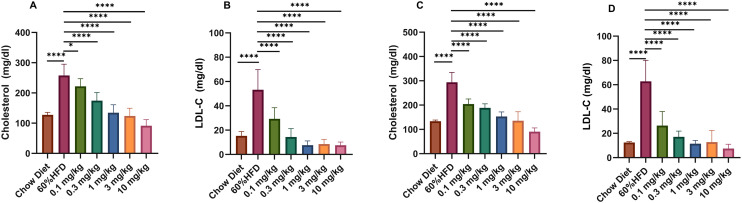
Plasma cholesterol and LDL-C levels. Plasma cholesterol and LDL-C levels in HFD-fed mice after 2 weeks of treatment with Kylo-0603 administration **(A and B)**. Plasma cholesterol and LDL-C levels in HFD-fed mice after 10 weeks of treatment with Kylo-0603 administration **(C and D)**. The data are shown as the means ± SDs; n = 5; P < 0.05, **P < 0.01, ***P < 0.001, and ****P < 0.0001 vs the high-diet control group according to one-way ANOVA with Dunnett’s post hoc test.

Specifically, after 2 weeks of treatment, mice treated with 3 mg/kg and 10 mg/kg Kylo-0603 presented 52.0% and 64.5% reductions in Chol levels (Fig 5A), respectively, and even more pronounced reductions in LDL-C levels of 84.0% and 85.8% (Fig 5B), respectively, than did the untreated HFD control group. Furthermore, at the end of the 10-week treatment, all the treatments (0.1 mg/kg, 0.3 mg/kg, 1 mg/kg, 3 mg/kg, and 10 mg/kg) significantly inhibited the Chol and LDL-C levels in a dose-dependent manner. Compared with those in the HFD control group, the Chol levels were reduced by 30.6%, 35.9%, 47.9%, 53.9%, and 69.2%, respectively (Fig 5C), whereas the reduction in LDL-C levels was even more significant at 58.0%, 72.7%, 81.8%, 79.7%, and 88.2%, respectively (Fig 5D). At the end of the 2nd and 10th weeks of pharmacological intervention, the remaining 5 mice in each group were subjected to a 6-hour fast, followed by euthanasia. The liver tissues from these mice were collected in isopropyl alcohol for homogenization. Following the intervention with Kylo-0603, alterations in Chol and TG concentrations in the liver were observed. The results demonstrated that Kylo-0603 did not exert a notable influence on the cholesterol content in liver homogenates. However, it was observed to be efficacious in reducing the triglyceride content. In particular, the reduction of triglycerides was most pronounced at a dose of 3 mg/kg, with an average reduction of up to 36.4% (see S10 Fig). These data not only reveal the potential of Kylo-0603 to regulate hepatic triglyceride levels under specific conditions, but also strongly demonstrate its potential value in regulating blood lipids and preventing hyperlipidemia and related cardiovascular diseases

### Efficacy of Kylo-0603 in MASH Mouse Models (F2 ~ 3)

**Fig 6:**
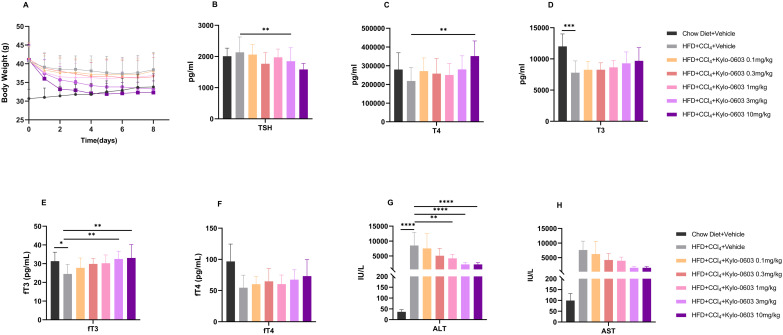
Pharmacological efficacy of Kylo-0603 in MASH mouse models (F2 ~ 3). A shows the body weights of the mice after 8 weeks of drug treatment. B-F show the levels of TSH, T3, T4, fT3, and fT4 in the blood of the mice in each group after 8 weeks of drug treatment. G-H Liver function tests (ALT and AST). The data are presented as the means ± SDs; *, p < 0. 05; **, p < 0.01; ***, p < 0.001; ****, p < 0.0001; one-way ANOVA with Dunnett’s post hoc test, n = 9.

Although the exact molecular pathogenesis of MASH remains to be elucidated, previous studies have shown that CCl_4_ can exacerbate liver fibrosis and damage induced by a high-fat diet (HFD; 60 kcal% fat) [41,42]. In light of these findings, the present study aimed to explore the potential protective effects of Kylo-0603 on changes in liver structure and function induced by the combination of CCl_4_ and a high-fat diet in 5-week-old male C57BL/6J mice. The experimental design was as follows: Mice were randomized into a control diet group and an HFD group, both of which received intraperitoneal injections of CCl_4_ at a dose of 0.2 ml/kg twice weekly. After 8 weeks of high-fat feeding and CCl_4_ dosing, the results showed that the mice in the CCl_4-_induced control group (HFD + CCl_4_ + Vehicle) developed significant hypothyroid-like symptoms, as evidenced by a significant decrease in the blood levels of T3, fT3, and fT4 and an increase in the TSH level. Moreover, the NAS (NAFLD Activity Score) was as high as 6.3, accompanied by significant inflammation (2.9), ballooning (1.8), and moderate to severe fibrosis (2.6, grades F2-3), meeting the diagnostic criteria for fibrotic MASH (Table 1). However, group with doses of 0.1 mg/kg, 0.3 mg/kg, 1 mg/kg, 3 mg/kg, or 10 mg/kg Kylo-0603 in combination with HFD and CCl_4_ solvent after received an 8-week treatment presented a significant reduction in the body weight of the mice, and this effect was dose-dependent (reductions ranging from 0.8% to 15.8%), in Comparison with the control group (HFD + CCl_4_ + Vehicle). In addition, total cholesterol and LDL-C levels were significantly reduced in a dose-dependent manner (the results of the experiment were similar to those described above for the administration of drugs in HFD-induced obese mice). More importantly, Kylo-0603 effectively reduced the abnormally elevated serum levels of ALT (Fig 6G) and AST (Fig 6H), particularly in the 3 mg/kg dose group, which presented the most significant effects, with ALT and AST decreasing by 73.8% and 76.3%, respectively

**Table 1:** NAS (NAFLD Activity Score) of liver tissue of mice in each group after 8 weeks of treatment with either vehicle (V) or Kylo-0603 (0.1-10 mg/kg/day).

Group	CD+ Vehicle	HFD + CCl4 ^+^Vehicle	HFD + CCl_4_ + Kylo-0603 0.1 mg/kg	HFD + CCl_4_ + Kylo-0603 0.3 mg/kg	HFD + CCl_4_ + Kylo-0603 1 mg/kg	HFD + CCl_4_ + Kylo-0603 3 mg/kg	HFD + CCl_4_ + Kylo-0603 10 mg/kg
Steatosis	0.9 ± 0.6	1.7 ± 0.9	0.8 ± 1.0*	0.3 ± 0.5***	0.1 ± 0.3****	0.2 ± 0.4****	0.4 ± 1.0***
Lobular Inflammation	0.3 ± 0.7****	2.9 ± 0.3	2.4 ± 0.5	2.2 ± 0.4*	2.2 ± 0.4*	1.4 ± 0.5****	1.1 ± 0.5****
Ballooning	0.0 ± 0.0****	1.8 ± 0.4	1.4 ± 0.5	1.4 ± 0.5	2.0 ± 0.0	1.1 ± 0.3*	1.0 ± 0.7**
“NAFLD Activity Score”	1.2 ± 0.8****	6.3 ± 1.0	4.7 ± 0.9**	4.0 ± 0.9****	4.3 ± 0.5****	2.8 ± 1.0****	2.6 ± 1.0****
Fibrosis Score	0.1 ± 0.3****	2.6 ± 0.5	2.8 ± 0.4	2.3 ± 0.5	2.3 ± 0.5	2.0 ± 0.0*	2.0 ± 0.0*

The data are presented as the means ± SDs; *, P < 0.05; **, P < 0.01; ***, P < 0.001; ****, P < 0.0001 vs control group the control group (HFD + CCl_4_ + Vehicle); one-way ANOVA with Dunnett’s post hoc test, n = 9.

It is noteworthy that after eight weeks of Kylo-0603 treatment in the MASH mouse models (F2 ~ 3), the levels of thyroid hormones T3, fT3, T4, and fT4 in the drug-treated group of mice exhibited increasing trends to varying degrees, while the TSH levels demonstrated decreasing trends to varying degrees (Fig 6B-F). These findings indicate that Kylo-0603 may facilitate partial improvement in hypothyroidism and mitigate MASH-related pathological alterations or damages in mouse hepatocytes (Fig 6G–H).

**Fig 7:**
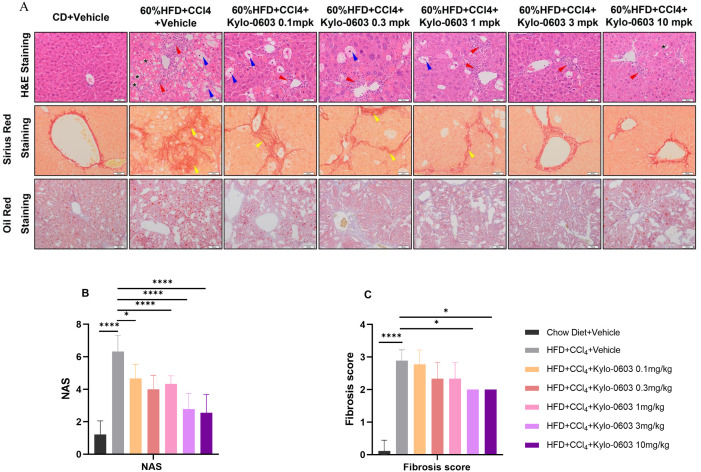
Hematoxylin and eosin (H&E) staining, Sirius Red staining, and Oil Red staining of liver tissues from mice in each group after 8 weeks of treatment with either vehicle or Kylo-0603 (0.1–10 mg/kg/day, as indicated). Photomicrographs of representative samples from each treatment group are shown. (*, lipid droplets; blue triangles, balloon-like changes; red triangles, inflammatory foci; yellow triangles, fibrosis) Scale bar = 50 μm, n = 9. A and B show the NAS (NAFLD Activity Scores) and fibrosis scores of the liver tissue of the mice after 8 weeks of drug intervention. The data are presented as the means ± SDs; *, p < 0. 05; **, p < 0.01; ***, p < 0.001; ****, p < 0.0001; one-way ANOVA with Dunnett’s post hoc test, n = 9.

The livers from the mice were harvested and evaluated for NAS (NAFLD Activity Score) and fibrosis using H&E staining, Sirius red staining, and quantitative real-time polymerase chain reaction (qRT-PCR). Following eight weeks of treatment with Ky-lo-0603, the mice in the drug-treated groups exhibited notable improvements in hepatic steatosis, inflammatory response, and ballooning degeneration in comparison to the control group (HFD + CCl_4_ + Vehicle), with no exacerbation of hepatic fibrosis. Specifically, at doses of 3 mg/kg and 10 mg/kg, mice exhibited notable reductions in hepatic steatosis and inflammation scores, exceeding 1.0 points (P < 0.001), with decreases in ballooning degeneration scores of 0.7 points (P < 0.05) and 0.8 points (P < 0.01), respectively (Table 1). Furthermore, the NAS (NAFLD Activity Score) of the two groups exhibited a notable decline, with a reduction of 3.5 points (P < 0.0001) and 3.7 points (P < 0.0001), respectively (Fig 7A). Additionally, the fibrosis score demonstrated a decrease of 0.6 points (P < 0.05) (Fig 7B and Table 1). These findings provide compelling evidence that Kylo-0603 not only ameliorates the pathological characteristics of MASH but also effectively inhibits further progression of liver fibrosis. The alterations in hepatic histology were correlated with reductions in plasma cholesterol and LDL-C. The same effect of H&E staining, Sirius red staining, and oil red staining of the liver was observed in HFD-induced obese mice (S11 Fig and S3 Table)

### Accurate assessment of the therapeutic effect of the Kylo-0603 drug via SHG (second harmonic generation)/TPEF (two-photon excitation fluorescence) microscopy technique in MASH mouse models (F2 ~ 3)

As a fundamental component of clinical prognosis in MASH, a precise evaluation of hepatic fibrosis is very crucial. Artificial intelligence-assisted second harmonic generation/two-photon excitation fluorescence (SHG/TPEF) microscopy technique has been introduced because the existing scoring systems are insufficient for capturing the process of hepatic fibrosis regression. This offers a consistent and very precise way to evaluate the pathological characteristics of MASH, especially the serial measurement of collagen fibers and hepatic fibrosis [43]. In this study, we employed second harmonic generation/two-photon excited fluorescence (SHG/TPEF) microscopy integrated with digital image analysis to evaluate liver tissue sections across a larger tissue area from MASH mouse models (F2–F3). This approach enabled systematic analysis of the effects of Kylo-0603 on arresting the progression of hepatic steatosis and fibrosis of liver tissue.

Through quantitative analysis of SHG/TPEF imaging datasets, we conducted a detailed assessment of the specific impacts of varying Kylo-0603 dosages (0.1 mg/kg, 0.3 mg/kg, 1 mg/kg, 3 mg/kg, and 10 mg/kg) on fibrogenesis and steatosis in F2–F3 MASH mouse models. The key findings are systematically summarized as follows:

a The assessment of fibrosis adopted a dual verification strategy combining traditional pathology and advanced artificial intelligence imaging technology, which consistently validated the ameliorative effect of Kylo-0603 (Table 1 and S12 Fig). Specifically, routine pathological scoring via Sirius Red Staining after 8 weeks of administration established a basic evaluation framework, while innovative TPE/SHG enabled precise quantification by: (1) specifically detecting type I/III collagen fibers (SHG signals) to eliminate false-positive interference from blood vessels/basement membranes; (2) achieving spatial quantification of lobular and bridging fibrosis through 3D positioning analysis of portal space (PS), central vein (CV), and portal tract (PT). Building upon our multimodal assessment strategy, TPE/SHG imaging analysis demonstrated that all Kylo-0603 treatment groups exhibited a dose-dependent and statistically significant reduction in hepatic fibrosis compared to the untreated control (HFD + CCl₄). Quantitative analysis revealed a dose-dependent decrease in fibrous tissue area, with fibrosis percentages decreasing from 4.36% in the control group to 2.92% (0.1 mg/kg), 2.12% (0.3 mg/kg), 2.16% (1 mg/kg), 2.03% (3 mg/kg), and 1.25–1.86% (10 mg/kg) across treatment cohorts. These intergroup differences were confirmed to be statistically significant (P < 0.05), demonstrating the therapeutic efficacy of Kylo-0603 in ameliorating hepatic fibrogenesis.
b Regarding steatosis, Kylo-0603 showed statistically significant improvements between dose groups (P < 0.05) and a substantial reduction in the area of steatosis when compared to the control group at all tested doses. Notably, steatosis was reduced by roughly 60% in the group receiving the lowest dose (0.1 mg/kg), and by a considerable 90% in the group receiving the highest dose (10 mg/kg). These results show the effective of Kylo-0603 in preventing steatosis. Specifically, the percentages of steatosis were 21.66% (HFD + CCl_4_), 5.07% (0.1 mg/kg), 4.39% (0.3 mg/kg), 4.19% (1 mg/kg), 2.22% (3 mg/kg), and 1.25% (10 mg/kg), respectively.

### Gene expression analysis in the liver of the MASH mouse models (F2 ~ 3)

**Fig 8:**
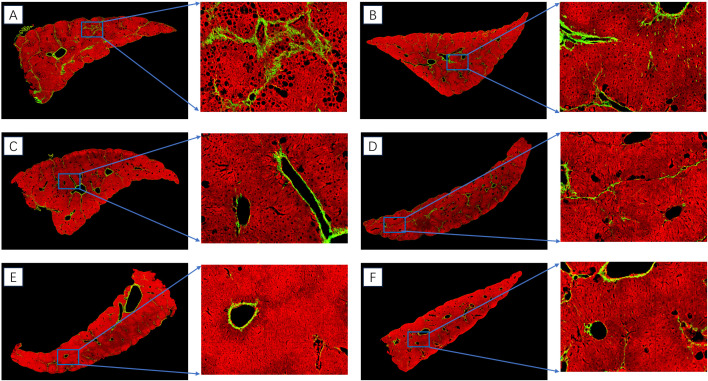
Effect of Kylo-0603 drug on the reversal of steatosis and inhibition of fibrotic process in liver tissues of the MASH mouse models (F2 ~ 3), analyzed by SHG/TPEF microscopy combined with digital image analysis technique. A shows the control and untreated groups (SHG fibrosis (green): 4.36%, steatosis (black lipid cavity and surrounding involved area):21.66%); B shows the sample image after 0.1 mg/kg administration (SHG fibrosis (green): 2.92%, steatosis (black lipid cavity and surrounding involved area):5. 07%); C shows the sample image after 0.3 mg/kg administration (SHG fibrosis (green): 2.12%, steatosis (black lipid cavity and surrounding involved area): 4.39%; D shows the sample after 1 mg/kg administration (SHG fibrosis (green): 2.16%, steatosis (black fat space and surrounding area): 4.19%); E shows the specimen after 3 mg/kg administration (SHG fibrosis (green): 2.03%, steatosis (black fat vacuoles and surrounding areas): 2.22%; F shows the sample after 10 mg/kg administration (SHG fibrosis (green): 1.86%, steatosis (black fat vacuoles and surrounding areas): 1.25%.

**Fig 9:**
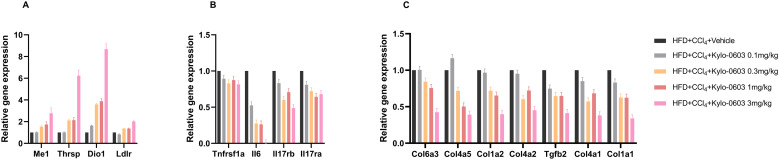
Effects of Kylo-0603 on the expression of related genes of the MASH mouse models. Total RNA was extracted from the frozen liver tissue of the MASH mouse models (F2 ~ 3), and the mice were injected daily with saline or Kylo-0603 at a dosage of 0.1–3 mg/kg/day. Transcriptome sequencing is based on the Illumina sequencing platform. The relative expression of a gene was calculated by normalizing the true expression level of the target gene to the expression level of that gene in the control group. A shows the trend of metabolism-related gene expression changes in liver tissue. B shows the inhibitory effect of Kylo-0603 on the inflammatory factor genes Tnfrsf1a, Tnfrsf11a, IL6, IL1b, IL17rb, IL17ra and Cxcl2. C shows the expression of collagen synthesis-related genes in liver tissue.

To explore the mechanism by which Kylo-0603 promotes lipid metabolism, we used Illumina’s NEBNext® UtraTM RNA Library Prep Kit to examine the expression of relevant genes in the livers of the MASH mouse models (F2 ~ 3) after Kylo-0603 administration (Fig 8). We first examined the expression of THR-regulated genes, including iodothyronine deiodinase 1 (Dio1), malic enzyme 1 (Me1), and thyroid hormone responsive (Thrsp). Me1 encodes an enzyme that catalyzes the conversion of malate to pyruvate while concomitantly generating NADPH from NADP, and its upregulation promotes energy metabolism [44]. Thrsp is mainly a nuclear protein induced by thyroid hormones, carbohydrate intake, adipose tissue differentiation and lactation and plays an important role in the regulation of lipid metabolism. The Dio1 gene in liver tissue is responsible for the conversion of T4 to T3, thereby facilitating intrahepatic energy metabolism and T3 production. Reduced DIO1 levels and activity have been observed in humans and rodents with advanced MASH, and DIO1 knockdown results in increased hepatic lipid content, suggesting that downregulation of DIO1 may exacerbate hepatic lipid accumulation and MASH progression. Kylo-0603 significantly upregulated the expression of Dio1, Thrsp and Me1 in a dose-dependent manner in the MASH mouse models (F2 ~ 3) (Fig 9A). LDL-R (low-density lipoprotein receptor) promotes the cellular uptake of LDL and facilitates cholesterol degradation. Kylo-0603 upregulated LDL-R gene expression in a dose-dependent manner. This finding was consistent with a dose-dependent reduction in the serum LDL-C level (Fig 9A). Kylo-0603 significantly downregulated the expression levels of genes closely related to inflammatory factors in liver tissues, which in turn effectively reversed the inflammatory response in liver tissues and significantly improved the pathology of MASH syndrome. This therapeutic effect was validated by assessing liver histopathology in animal models. Kylo-0603 inhibited the genes encoding the inflammatory factors Tnfrsf1a, IL6, IL17rb, and IL17ra (Fig 9B).

We thoroughly analyzed the dynamics of gene expression in liver tissues closely related to fibrosis, focusing on collagen synthesis genes that play a central role in the pathological process of fibrosis (see S13–S17 Fig). The results revealed that the expression of these genes was significantly suppressed with increasing doses of the Kylo-0603 drug, demonstrating a distinct dose-dependent characteristic (Fig 9C). These findings directly demonstrate that Kylo-0603 can effectively reduce abnormal collagen deposition in the liver by regulating collagen synthesis. This mechanism not only provides intuitive evidence for the active role of the drug in the treatment of liver fibrosis but also strongly supports its potential to alleviate or even stop the fibrotic process.

Notably, Kylo-0603 stimulated the upregulation of peroxisome-related genes in a dose-dependent manner. These results demonstrated the unique ability of Kylo-0603 to improve MASH symptoms, which may be largely attributed to its unique liver-targeting properties.

## Discussion

MASLD is currently the most common liver disease worldwide and a major cause of liver-related morbidity and mortality. According to statistics, the prevalence of MASLD has reached as high as 38% [45], and it is still rising at a startling rate. Because of its intricate pathophysiology, MASH, a severe manifestation of MASLD, presents substantial obstacles to the advancement of clinical studies and the creation of new medications. Currently, only one MASH drug, Resmetirom, was approved for marketing in the United States on March 14th this year, primarily for the treatment of adults with noncirrhotic MASH with moderate to advanced liver fibrosis (consistent with stages F2 to F3 fibrosis) in the USA [46]. In vitro THR competition assays demonstrated that Resmetirom exhibited the highest degree of THR-β selectivity among thyromimetic agents, with a selectivity ratio of 12 or 17 for THR-β over THR-α. GC-1 exhibited a selectivity ratio of approximately 0.9 to 10 for THR-β over THR-α, while VK2908A (MB07344) demonstrated a selectivity ratio of 2.5 to 15.8 for THR-β over THR-α. These findings were previously published [28,47,48,49]. In this work, we developed the drug Kylo-0603, which, although not optimal in terms of THRβ selectivity, is endowed with unprecedented liver-targeting properties owing to its unique GalNAc structural design [38,50]. This innovative design ensures that the drug can be efficiently and specifically transported to the liver, significantly reducing potential side effects on other nontarget tissues in the body, with drug concentrations in the liver measured to be nearly 70,000 times higher than those in the blood at 0.25 h after a single dose in Rats. For example, the tissue/plasma ratios of GC-1 and T3 in the liver are similar [40]. Owing to its unique high hepatic targeting ability, Kylo-0603 shows exceptional properties in treating MASH disease.

Obesity is an important medical challenge that not only affects the quality of life of individuals but also significantly increases morbidity and mortality from many diseases. Current treatments for obesity are effective but still have significant limitations in terms of safety and efficacy, which has prompted researchers to explore new therapeutic strategies continuously. Among them, thyroid hormone receptor modulators have attracted much attention for their potential therapeutic effects. Thyroid hormones exert their physiological effects through two main receptors, TRα and TRβ. TRα mainly mediates the effects of thyroid hormones on the heart, whereas TRβ is strongly associated with the control of obesity and hypercholesterolemia, and its activation does not trigger the undesirable side effects of thyroid hormones on the heart, bones, or skeletal muscles. However, the action of endogenous thyroid hormones is nonselective and can lead to several adverse effects, including cardiac stimulation.

In this study, Kylo-0603, a novel drug, demonstrated significant potential for weight loss and lipid reduction. The drug effectively reduced the body weight of high-fat diet-induced obese mice, especially at doses of 3 mg/kg and 10 mg/kg. Moreover, Kylo-0603 also significantly reduced the amount of body fat in the mice, a mechanism similar to that previously studied for T3 and GC-1 weight reduction through increased oxygen consumption. However, Kylo-0603 is unique because of its ability to target the liver. This means that the drug acts primarily on TRβ in the liver can avoid the adverse effects that nonselective thyroid hormones or thyroid hormone-like structure compounds caused extrahepatic organs such as the heart. In addition, Kylo-0603 has unique advantages over the currently popular GLP-1-targeting drugs. Although GLP-1 drugs can control blood sugar and reduce body weight to a certain extent, their mechanism of action is relatively complex, and 40% of their body weight reduction is muscle volume [51,52]. In contrast, Kylo-0603 achieves more precise and effective weight loss and fat reduction effects by acting directly on THRβ in the liver and reducing only fat mass. Thyroid status strongly influences contractile function and the mass of skeletal muscle. However, Kylo-0603 has high liver-specific targeting ability and selectivity for THRβ, and it has been demonstrated to not cause T3-mediated fiber type shifts or nutrient changes related to bone mineral density (BMD/BMC) (S5 Fig).

In a high-fat diet-induced obese mouse model, we evaluated the effects of Kylo-0603 on lipid metabolism. The results demonstrated that after 2–10 weeks of Kylo-0603 intervention, Kylo-0603 significantly reduced serum total Chol and LDL-C levels in mice compared with HFD control group. Furthermore, this reduction exhibited a dose-dependent relationship. This finding is consistent with previous results with THR-β-selective agonists such as GC-1 and MGL-3196, both of which have similar effects. Notably, the Kylo-0603-induced alterations in lipid profiles were consistent with corresponding changes in the expression of genes regulating hepatic lipid metabolism (Fig 9). In particular, similar to previous studies showing that both T3 and GC-1 significantly increased the expression of Dio1 and Me1, the Kylo-0603 study yielded consistent results. In addition, the dose-dependent upregulation of carnitine palmitoyl transferase 1α (Cpt1a), a direct transcriptional target of THR-β [53], demonstrated a pronounced dose-dependent upregulation that correlates with enhanced mitochondrial β-oxidation capacity. Concomitantly, significant increases in pyruvate dehydrogenase kinase 4 (Pdk4) expression further substantiated THR-β-mediated activation of mitochondrial oxidative phosphorylation (S5 Fig). Complementing these findings, the coordinated downregulation of sterol regulatory element-binding protein 1c (Srebf1) and stearoyl-CoA desaturase 1 (Scd1) aligns mechanistically with THR-β’s established role in suppressing lipogenic pathways, while the dose-responsive elevation of adipose triglyceride lipase (Atgl) expression unequivocally demonstrates THR-β-driven potentiation of lipolytic processes [53]. Through comprehensive transcriptional monitoring of these pivotal regulatory nodes, our study elucidates an integrated therapeutic mechanism whereby THR-β agonism orchestrates three synergistic actions: 1) activation of mitochondrial fatty acid oxidation through Cpt1a-Mcad-Pdk4 axis potentiation, 2) suppression of de novo lipogenesis via Srebf1-Scd1 downregulation, and 3) enhancement of lipid mobilization through Atgl induction.

In addition, loss of peroxisomes as well as impaired peroxisomal functions have been demonstrated to occur in inflammatory conditions including MASH [54]. This study reveals that Kylo-0603 upregulates peroxisome-related genes (S17 Fig). This finding further confirms the unique effect of Kylo-0603 in alleviating MASH symptoms, primarily due to its distinctive hepatic targeting properties. For additional details regarding the expression of other relevant liver genes, please refer to the heatmap in the Supporting Material.

LDL-R is crucial to the metabolism of LDL particles. LDL-C combines with LDL-R on the surface of the liver cell membrane and enters human liver cells through endocytosis. In mice with HFD induced obesity models or MASH mouse models (F2 ~ 3), Ky-lo-0603 demonstrated a significant and dose-dependent reduction in cholesterol and LDL-C levels. This finding aligns with the mechanism by which thyroid hormones and thyroid hormone analogs reduce cholesterol levels by increasing the expression of hepatic LDL-R [48,55].

In addition to the aforementioned studies on body weight and lipids, we also investigated the impact of the Ky-lo-0603 drug on liver fibrosis. The results demonstrated that after 8 weeks of treatment with Kylo-0603, histological analysis via H&E staining revealed significant improvements in fat content, inflammation, and ballooning in liver tissues, indicating that Kylo-0603 ameliorated MASH symptoms without exacerbating liver fibrosis. To elucidate the pharmacodynamic properties of Kylo-0603 in alleviating MASH symptoms, we employed advanced two-photon excitation/second harmonic generation (TPE/SHG) imaging to quantitatively assess alterations in liver fibrosis. Our analysis revealed that Kylo-0603 demonstrated dose-dependent efficacy in MASH mouse models, significantly attenuating both hepatic steatosis and fibrosis progression. These findings underscore its potential as a multifaceted therapeutic agent for MASH and related liver diseases.

This paper presents a comprehensive examination of the expression of genes associated with lipid metabolism and fibrosis, along with an extensive analysis of the expression changes of numerous related genes. For instance, impaired peroxisome function is linked to the development of MASH [54]. This study reveals that Kylo-0603 upregulates peroxisome-related genes. This finding further confirms the unique effect of Kylo-0603 in alleviating MASH symptoms with the adverse effects commonly observed with other THR-β agonists primarily due to its distinctive hepatic targeting properties. For additional details regarding the expression of other relevant liver genes, please refer to the heatmap in the Supporting Material (S13–S17 Fig).

Notably, T3-like binding to THR-α of THR-β agonist has been demonstrated to exert deleterious effects on the skeletal and cardiac systems, which represents a significant impediment to its utilization in the treatment of obesity and MASH. The novel THR-β agonist Kylo-0603, developed in this study, can specifically target the liver (S4 Table), thereby effectively circumventing the potential adverse effects that may be induced by nonspecific binding of THRα in cardiac tissues. In the MASH mouse model induced, treatment with Kylo-0603 resulted in significant improvement in hypothyroidism and a notable reduction in abnormally elevated levels of alanine aminotransferase (ALT) and alanine aminotransferase (AST) (Fig 6). Furthermore, this effect was dose-dependent. In conclusion, these data demonstrate that Kylo-0603 is an effective agent for reducing the degree of hepatic injury and improving hypothyroidism induced by CCl_4_ injection.

To evaluate the preclinical safety profile of Kylo-0603, a novel liver-targeted thyroid hormone receptor β (TRβ) agonist with therapeutic potential, systematic toxicological assessments were conducted in both non-human primates (cynomolgus monkeys) and rodents (Sprague-Dawley rats). In a 28-day repeated-dose toxicity study involving oral administration of Kylo-0603 to cynomolgus monkeys at doses of 0.5, 1, and 5 mg/kg per day, followed by a 56-day recovery period, no macroscopic lesions or histopathological changes attributable to Kylo-0603 were observed during or after the dosing or recovery phases. This phenomenon was also observed in the rodent model: the 182-day long-term dosing study in Sprague-Dawley rats (with a maximum dose of 1.5 mg/kg/day and an 8-week recovery period) showed only mild Kylo-0603-related pathological changes. The no-observed-adverse-effect level (NOAEL) was determined to be 1.5 mg/kg/day. Collectively, these independent toxicological investigations substantiate Kylo-0603’s favorable safety, providing rigorous preclinical support for clinical advancement. Critically, no perturbations of the hypothalamic-pituitary-thyroid (HPT) axis were identified in either species.

In conclusion, this study designed and synthesized an innovative liver-targeting compound, Kylo-0603, with unique both liver-targeting and THR-β agonist properties. Kylo-0603 was crafted through precise chemical design, connecting three galactosamine structures to a T3-like structure via specific ester or amide bonds, resulting in an efficient liver-targeting molecule. Experimental validation demonstrated that Kylo-0603 exhibits exceptional stability in plasma, high selectivity for THRβ, and excellent pharmacokinetic and safety characteristics. This research breakthrough not only opens up a new avenue for exploring therapeutic drugs for MASLD and MASH but also lays a solid scientific foundation for potentially significant breakthroughs in improving the quality of life for patients with liver diseases in the future.

## Supporting information

S1 Fig The Laboratory Animal Flow Diagram. (TIF)

S2 Fig General procedure for the synthesis of A and A-c1. Synthesis of **A-c1**: To a solution of dlSANCc12(84 mg) and cbz-6-aminocaproic acid(24 mg) in DMF (8 ml), was add HOBt(21.6 mg) and DIPEA(53.5 mg) at 0 °C. After stirring at room temperature for 16 h, the reaction mixture quenched with water, and was then extracted with DCM (20 mL x 3). The organic phase was washed with brine (20 mL x 3) and dried over NaSO_4_. After removal of the solvent under reduced pressure, the crude product was purified by chromatography to give the desired product **A-c1**(72.8 mg) as a white solid. Synthesis of **A**: To a solution of A-C1(72.8 mg) in CH_3_OH (15 ml), was add Pd/C (3.4 mg) under a hydrogen atmosphere. Stirring of the resulting mixture was continued for 1.0 h at 40 °C, and Pd/C is removed through filtration. The solvent was then evaporated under reduced pressure to obtain 47 mg white solid. The characterization data of A are consistent with the previous reports.
(TIF)

S3 Fig General procedure for the synthesis of B. Step 1: To a solution of B-C3(36.1 mg, 0.10 mmol, 1.0 equiv) in THF(60 ml), was added 1.0 mol/L n-BuLi in N-hexane solution. After 3 h of reaction, a THF solution (5 mL) of compound B-c2 (33.4 mg, 0.13 mmol, 1.3 equiv) was added dropwise for another 3 h. The reaction was quenched with saturated ammonium chloride (20 mL), extracted with ethyl acetate (20 mL), the organic phase was washed with pyridine (30 mL), dried over NaSO4, and compound B-c4 (54.3 mg) was obtained by chromatography. Step 2: To a solution of B-C4 (54.3 mg, 0.10 mmol, 1.0 equiv)) in THF (50 mL), 1.0 mol/L TBAF solution (3 mL) was added. Completion of the reaction was monitored by TLC. The reaction mixture was quenched with water and then extracted with ethyl acetate (50 mL). The organic phase was washed with brine (20 mL) and pyridine (20 mL) and dried over NaSO4. After removal of the solvent under reduced pressure, the crude product was purified by chromatography to give a white solid (23.3 mg). To a solution of the above white solid in DMF (5 mL), cesium carbonate (40.4 mg) and benzyl bromoacetate (15.9 mg) were added at 0°C. After reaction at 40°C for 4 h, the solution obtained was diluted with MTBE (10 mL), filtered, water (20 mL) was added, then the aqueous phase was extracted with MTBE (20 mL*2). The organic phase was washed with brine (20 mL) and pyridine (20 mL) and dried over NaSO4. After removal of the solvent under reduced pressure, the crude product was purified by chromatography to give a white solid, which was dissolved in acetic acid (5 mL), catalyst 10% Pd/C (0.2 g) was added, hydrogenated overnight at room temperature, filtered, spin evaporated and column chromatographed to give a pale yellow solid compound B (15 mg). The characterization data of B are in agreement with the previous reports.
(TIF)

S4 Fig General procedure for the synthesis of Kylo-0603. The specific experimental operation was to add DMF (3.0 mL), compound B (15 mg), TBTU (8.47 mg) and DIPEA (20.2 mg) sequentially to the reaction vial and react for 6 h. Then compound A (47 mg) was added rapidly and stirred at room temperature for 2 h. The reaction was detected by HPLC, and the reaction was completed and terminated. The reaction solution was prepared with 1.0 mol/L ammonia solution under ice bath conditions to make the pH of the reaction solution 8−10. The ice bath was removed and the reaction was stirred at room temperature for half an hour while HPLC detection was performed. After the reaction was completed, the pH was adjusted to 7.0 with glacial acetic acid and then concentrated. The concentrated residue was dissolved with 35% acetonitrile/water, filtered and lyophilized to obtain 29.47 mg of the target compound. 1H NMR (400 MHz, DMSO-d6) δ 8.97 (s, 1H), 8.01 (d, J = 5.2 Hz, 1H), 7.82 (t, J = 5.2 Hz, 3H), 7.65 (d, J = 8.8 Hz, 3H), 7.00 (s, 1H), 6.82 (s, 1H), 6.65 (s, 2H), 6.61 (d, J = 8.0 Hz, 1H), 6.45 (d, J = 7.6 Hz, 1H), 4.61 (t, J = 5.2 Hz, 3H), 4.57 (d, J = 4.8 Hz, 3H), 4.48 (d, J = 2.8 Hz, 3H), 4.40 (s, 2H), 4.24 (d, J = 8.0 Hz, 3H), 3.79 (s, 2H), 3.73 (m, 3H), 3.69 (m, 3H), 3.53 (m, 24H), 3.34 (m, 6H), 3.12 (m, 3H), 3.03(d, J = 5.2 Hz, 6H), 2.29 (t, J = 5.6 Hz, 6H), 2.15 (s, 6H), 2.07 (t, J = 7.2 Hz, 3H), 1.82 (s, 9H), 1.24–1.43 (m, 30H), 1.09 (d, J = 6.8 Hz, 6H). 13C NMR (100 MHz, DMSO-d6)) 172.98, 170.57, 170.09, 168.23, 155.91, 152.70, 138.12, 134.35, 130.92, 130.28, 125.88,125.35, 115.28, 114.52, 101.84, 75.65, 71.98, 68.79, 68.54, 68.05, 67.83, 67.37, 60.99, 59.98, 52.64, 38.99, 38.69, 36.46, 36.34, 33.58, 29.63, 29.53, 29.36, 26.91, 26.69, 26.40, 25.66, 25.50, 23.47, 22.93, 20.51; HR-MS (ESI) m/z calcd for C81H134N8O28 [M + H] +: 1667.9380, found: 1667.9382.
(TIF)

S5 Fig ^1^H NMR spectra for kylo-0603. (TIF)

S6 Fig ^13^C NMR spectra for Kylo-0603. (TIF)

S7 Fig ESI-HRMS spectra for Kylo-0603. (TIF)

S8 Fig The body weight and cholesterol levels of the mice were recorded before the administration of the drug. Changes in body weight of mice in each group before drug administration during 16 weeks of how diet group and high-fat diet (A); The body weight, cholesterol levels, and LDL-c levels of the mice were recorded before the administration of the Kylo-0603 (B-D); The dates were shown in Mean ± SD, n = 16 (Chow diet group) & 96 (HFD group). The body weight and cholesterol levels of the mice were recorded before the administration of the drug, both in the control group and during the period of feeding on a high-fat diet.
(TIF)

S9 Fig After 8 weeks of treatment, body fat analysis and bone mineral density testing of mice in each group. A is the value of the length of the mouse body; B is the lean mass of mice; C is the bone mineral density of mice; D is the bone mineral content of mice. The data are shown as the means ± SDs; n = 5; P < 0.05, **P < 0.01, ***P < 0.001, and ****P < 0.0001 vs the high-diet control group according to one-way ANOVA with Dunnett’s post hoc test.
(TIF)

S10 Fig The Chol and TG content in liver homogenates of the mice in each group after 2 and 10 weeks of drug intervention. A is the Chol content in liver homogenates of the mice in each group after 2 and 10 weeks of drug intervention; Bis the TG content in liver homogenates of mice in each group after 2 and 10 weeks of drug intervention. The data are shown as the means ± SDs; n = 5; P < 0.05, **P < 0.01, ***P < 0.001, and ****P < 0.0001 vs the high-diet control group according to one-way ANOVA with Dunnett’s post hoc test.
(TIF)

S11 Fig DIO Mice Liver Staining. H&E staining, Sirius red staining, and oil red staining of the liver in a diet-induced obese (DIO) mouse model in each group after 8 weeks of pharmacological intervention; steatosis (*), inflammatory foci (red arrows), fibrosis (yellow triangles) and ballooning (blue arrows). Scale bar = 50 μm. n = 8.
(TIF)

S12 Fig The Fibrosis scoring by Sirius Red staining and SHG/TPEF. A: Conventional Histopathological Assessment: Fibrosis staging across experimental groups is demonstrated via Sirius Red staining in MASH mouse models (F2 ~ 3). B: SHG/TPEF-Based Fibrosis Quantification: Fibrosis scoring is performed using second harmonic generation/two-photon excitation fluorescence (SHG/TPEF) imaging in MASH mouse models.
(TIF)

S13 Fig The trends of cholesterol metabolism-related gene expression in liver tissue. Following a 10-week administration of Kylo-0603 to the HFD + CCl4-induced MASH mouse model, a comparison was conducted between the HFD control group and observed trends in gene expression in liver and cardiac tissue. These trends were related to energy metabolism, lipid accumulation, and hepatic histopathology (covering inflammation and fibrosis) and cardiac function. The aforementioned trends are illustrated in the heatmap displayed below. The figure depicts the dosing groups represented by each symbol, which are as follows: G1 (HFD control), G2 (0.1 mg/kg Kylo-0603), G3 (0.3 mg/kg Kylo-0603), G4 (1 mg/kg Kylo-0603), and G5 (3 mg/kg Kylo-0603). The heatmap illustrates the expression of genes associated with liver tissue, with blue representing low expression and red representing high expression.
(TIF)

S14 Fig The trends of gene expression in the metabolism of triglycerides in liver tissue. (TIF)

S15 Fig The trends in the expression of inflammation-related genes in liver tissue. (TIF)

S16 Fig The trends in the expression of fibrosis-related genes in liver tissue. (TIF)

S17 Fig Trends of peroxisome-related gene expression in liver tissue. (TIF)

S1 Table The the administration schedule for the different experimental groups. (DOCX)

S2 Table Detailed drug administration and preparation instructions for each experimental group. Administration volume: 10 μL per gram of body weight (10 μL/g × body weight in grams).
(DOCX)

S3 Table Results of reading scores of DIO mouse liver histopathologic test results in each group after 8 weeks of drug intervention, n = 8. (DOCX)

S4 Table Tissue distribution of Kylo-0603. The detailed experimental design and results are as follows: After a single oral administration of 12.5 mg/200 μCi/kg [^14^C] Kylo-0603 to SD rats (n = 6, 3 males and 3 females per group), the compound in tissues were detected by semi-quantitive radioactivities from 0 to 168 hours. The experiment results showed that 94.2% of the radioactivity was excreted via feces, with only 0.05% excreted in urine. For the tissue distribution study, the same dosing regimen was used to measure the amount of radioactivity in various tissues at 0.083, 0.25, 1, 4 and 24 hours (n = 6; three females and three males at each time point). The data showed that the highest concentrations of radioactivity were found in the liver, followed by the stomach, small intestine and large intestine. Very low levels were found in the spleen, kidney, lung and adipose tissue. Concentrations in bone, the thyroid gland, the thymus gland, the heart, skeletal muscle and the whole brain were all below the limit of detection. These data collectively confirm the significant liver-targeting property of Kylo-0603.
(DOCX)
