# Supplementary figures and images for: KYLO-0603, a novel liver-targeting, thyroid hormone receptor-β agonist for the inhibition of MASH progression
Source: PLoS One. 2025 Sep 15;20(9):e0331768. doi: 10.1371/journal.pone.0331768 (PMC12435690; doi:10.1371/journal.pone.0331768)

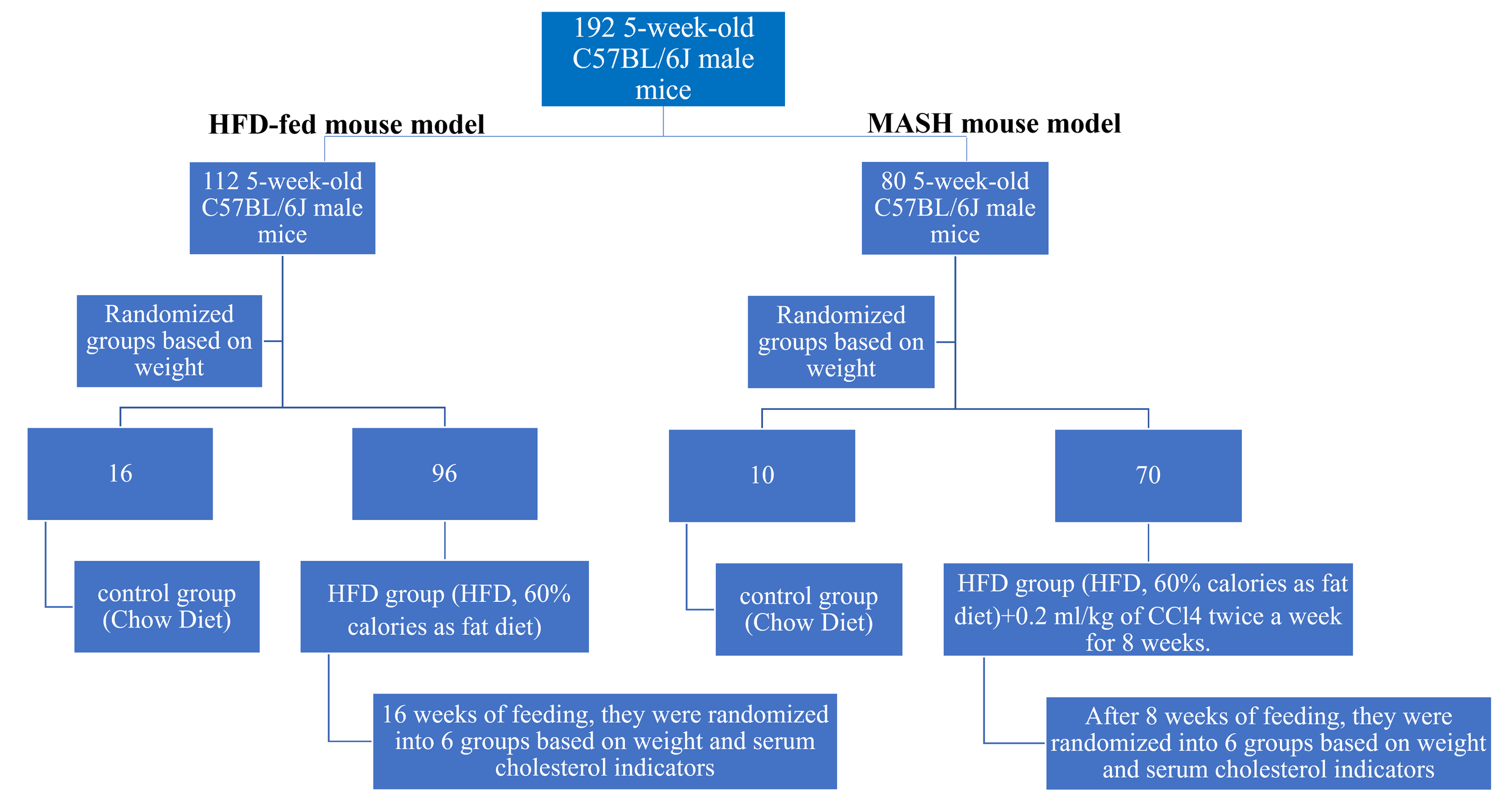

Supplement: S1 Fig — (TIF) [file pone.0331768.s001.tif]

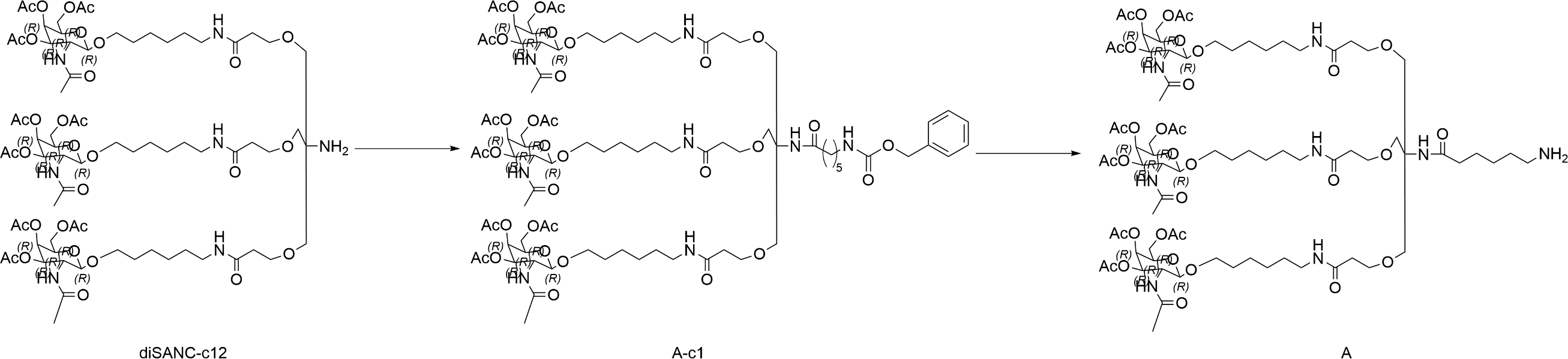

Supplement: S2 Fig — Synthesis of A-c1: To a solution of dlSANCc12(84 mg) and cbz-6-aminocaproic acid(24 mg) in DMF (8 ml), was add HOBt(21.6 mg) and DIPEA(53.5 mg) at 0 °C. After stirring at room temperature for 16 h, the reaction mixture quenched with water, and was then extracted with DCM (20 mL x 3). The organic phase was washed with brine (20 mL x 3) and dried over NaSO4. After removal of the solvent under reduced pressure, the crude product was purified by chromatography to give the desired product A-c1(72.8 mg) as a white solid. Synthesis of A: To a solution of A-C1(72.8 mg) in CH3OH (15 ml), was add Pd/C (3.4 mg) under a hydrogen atmosphere. Stirring of the resulting mixture was continued for 1.0 h at 40 °C, and Pd/C is removed through filtration. The solvent was then evaporated under reduced pressure to obtain 47 mg white solid. The characterization data of A are consistent with the previous reports. (TIF) [file pone.0331768.s002.tif]

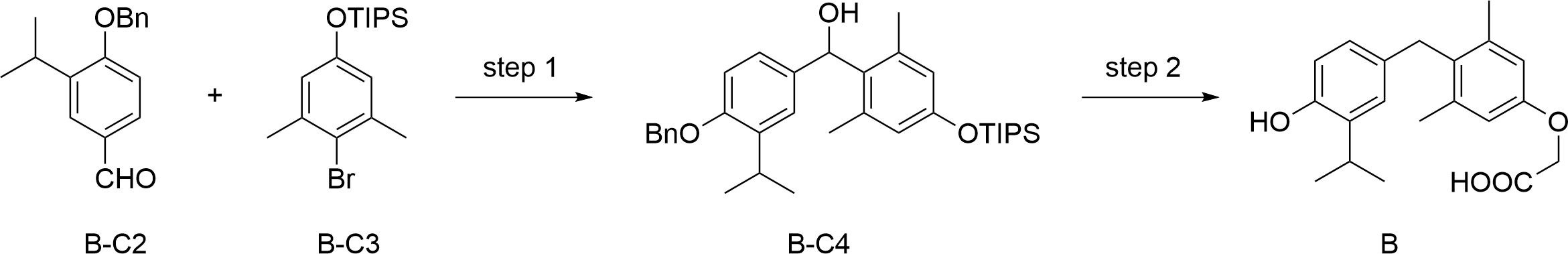

Supplement: S3 Fig — Step 1: To a solution of B-C3(36.1 mg, 0.10 mmol, 1.0 equiv) in THF(60 ml), was added 1.0 mol/L n-BuLi in N-hexane solution. After 3 h of reaction, a THF solution (5 mL) of compound B-c2 (33.4 mg, 0.13 mmol, 1.3 equiv) was added dropwise for another 3 h. The reaction was quenched with saturated ammonium chloride (20 mL), extracted with ethyl acetate (20 mL), the organic phase was washed with pyridine (30 mL), dried over NaSO4, and compound B-c4 (54.3 mg) was obtained by chromatography. Step 2: To a solution of B-C4 (54.3 mg, 0.10 mmol, 1.0 equiv)) in THF (50 mL), 1.0 mol/L TBAF solution (3 mL) was added. Completion of the reaction was monitored by TLC. The reaction mixture was quenched with water and then extracted with ethyl acetate (50 mL). The organic phase was washed with brine (20 mL) and pyridine (20 mL) and dried over NaSO4. After removal of the solvent under reduced pressure, the crude product was purified by chromatography to give a white solid (23.3 mg). To a solution of the above white solid in DMF (5 mL), cesium carbonate (40.4 mg) and benzyl bromoacetate (15.9 mg) were added at 0°C. After reaction at 40°C for 4 h, the solution obtained was diluted with MTBE (10 mL), filtered, water (20 mL) was added, then the aqueous phase was extracted with MTBE (20 mL*2). The organic phase was washed with brine (20 mL) and pyridine (20 mL) and dried over NaSO4. After removal of the solvent under reduced pressure, the crude product was purified by chromatography to give a white solid, which was dissolved in acetic acid (5 mL), catalyst 10% Pd/C (0.2 g) was added, hydrogenated overnight at room temperature, filtered, spin evaporated and column chromatographed to give a pale yellow solid compound B (15 mg). The characterization data of B are in agreement with the previous reports. (TIF) [file pone.0331768.s003.tif]

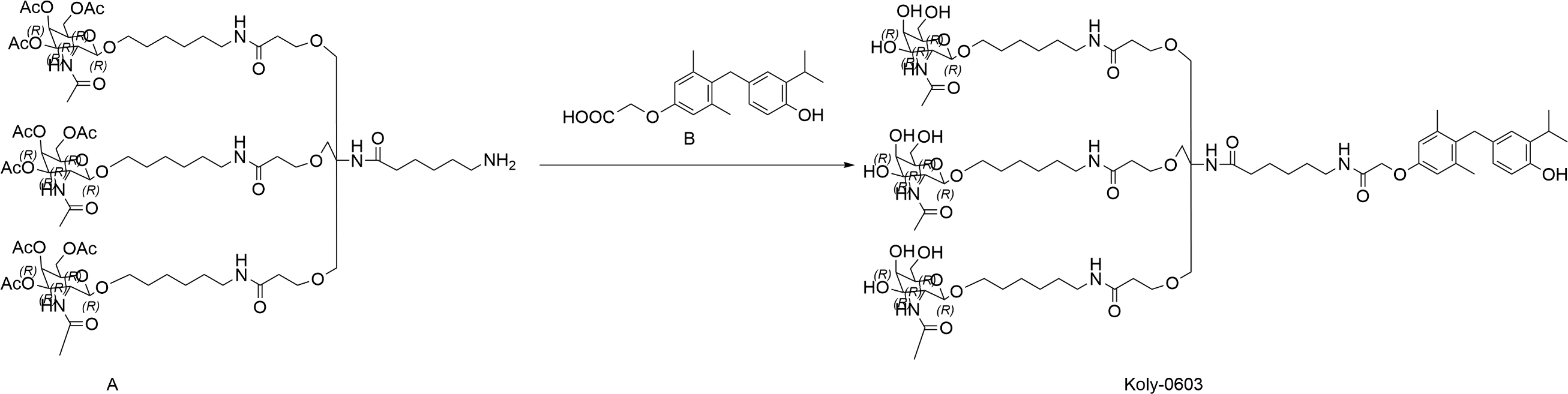

Supplement: S4 Fig — The specific experimental operation was to add DMF (3.0 mL), compound B (15 mg), TBTU (8.47 mg) and DIPEA (20.2 mg) sequentially to the reaction vial and react for 6 h. Then compound A (47 mg) was added rapidly and stirred at room temperature for 2 h. The reaction was detected by HPLC, and the reaction was completed and terminated. The reaction solution was prepared with 1.0 mol/L ammonia solution under ice bath conditions to make the pH of the reaction solution 8−10. The ice bath was removed and the reaction was stirred at room temperature for half an hour while HPLC detection was performed. After the reaction was completed, the pH was adjusted to 7.0 with glacial acetic acid and then concentrated. The concentrated residue was dissolved with 35% acetonitrile/water, filtered and lyophilized to obtain 29.47 mg of the target compound. 1H NMR (400 MHz, DMSO-d6) δ 8.97 (s, 1H), 8.01 (d, J = 5.2 Hz, 1H), 7.82 (t, J = 5.2 Hz, 3H), 7.65 (d, J = 8.8 Hz, 3H), 7.00 (s, 1H), 6.82 (s, 1H), 6.65 (s, 2H), 6.61 (d, J = 8.0 Hz, 1H), 6.45 (d, J = 7.6 Hz, 1H), 4.61 (t, J = 5.2 Hz, 3H), 4.57 (d, J = 4.8 Hz, 3H), 4.48 (d, J = 2.8 Hz, 3H), 4.40 (s, 2H), 4.24 (d, J = 8.0 Hz, 3H), 3.79 (s, 2H), 3.73 (m, 3H), 3.69 (m, 3H), 3.53 (m, 24H), 3.34 (m, 6H), 3.12 (m, 3H), 3.03(d, J = 5.2 Hz, 6H), 2.29 (t, J = 5.6 Hz, 6H), 2.15 (s, 6H), 2.07 (t, J = 7.2 Hz, 3H), 1.82 (s, 9H), 1.24–1.43 (m, 30H), 1.09 (d, J = 6.8 Hz, 6H). 13C NMR (100 MHz, DMSO-d6)) 172.98, 170.57, 170.09, 168.23, 155.91, 152.70, 138.12, 134.35, 130.92, 130.28, 125.88,125.35, 115.28, 114.52, 101.84, 75.65, 71.98, 68.79, 68.54, 68.05, 67.83, 67.37, 60.99, 59.98, 52.64, 38.99, 38.69, 36.46, 36.34, 33.58, 29.63, 29.53, 29.36, 26.91, 26.69, 26.40, 25.66, 25.50, 23.47, 22.93, 20.51; HR-MS (ESI) m/z calcd for C81H134N8O28 [M + H] +: 1667.9380, found: 1667.9382. (TIF) [file pone.0331768.s004.tif]

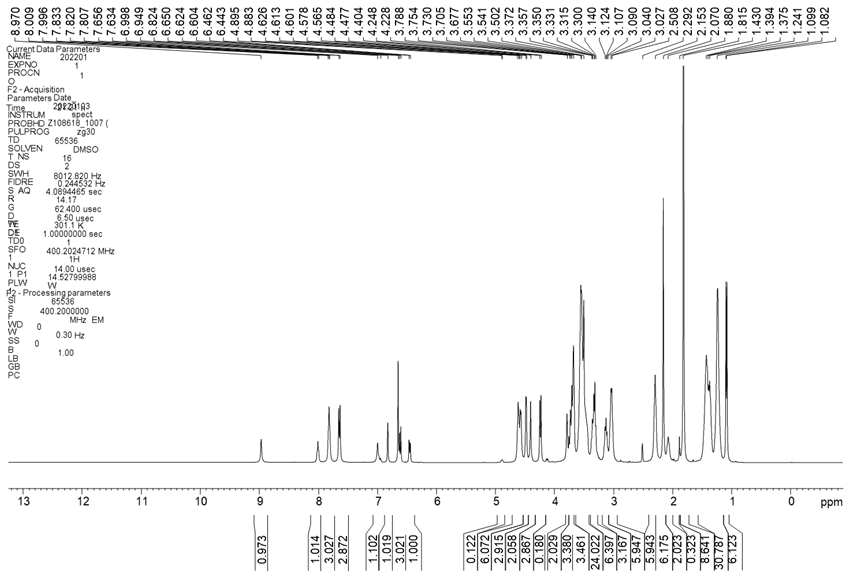

Supplement: S5 Fig — (TIF) [file pone.0331768.s005.tif]

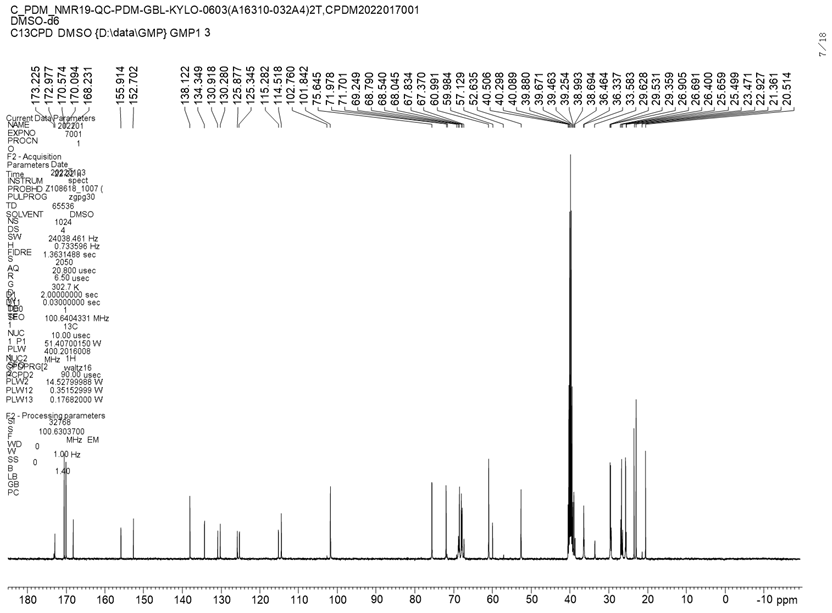

Supplement: S6 Fig — (TIF) [file pone.0331768.s006.tif]

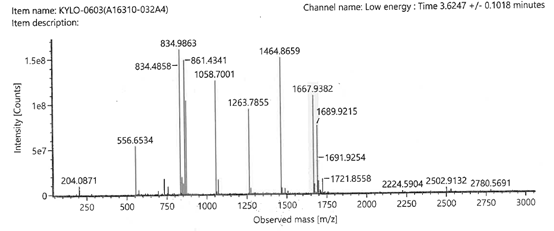

Supplement: S7 Fig — (TIF) [file pone.0331768.s007.tif]

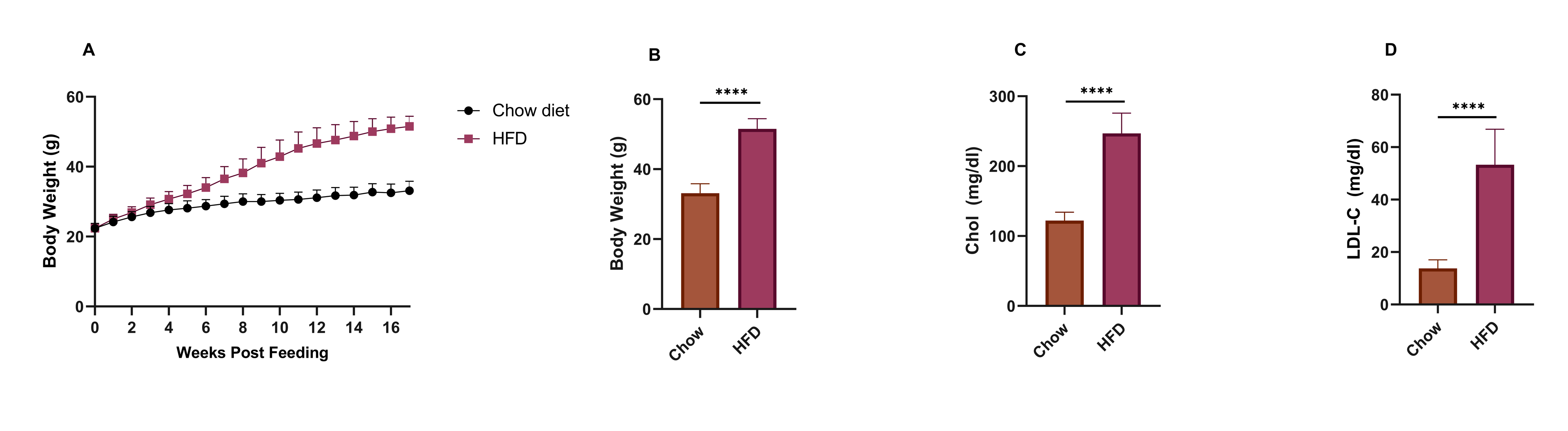

Supplement: S8 Fig — Changes in body weight of mice in each group before drug administration during 16 weeks of how diet group and high-fat diet (A); The body weight, cholesterol levels, and LDL-c levels of the mice were recorded before the administration of the Kylo-0603 (B-D); The dates were shown in Mean ± SD, n = 16 (Chow diet group) & 96 (HFD group). The body weight and cholesterol levels of the mice were recorded before the administration of the drug, both in the control group and during the period of feeding on a high-fat diet. (TIF) [file pone.0331768.s008.tif]

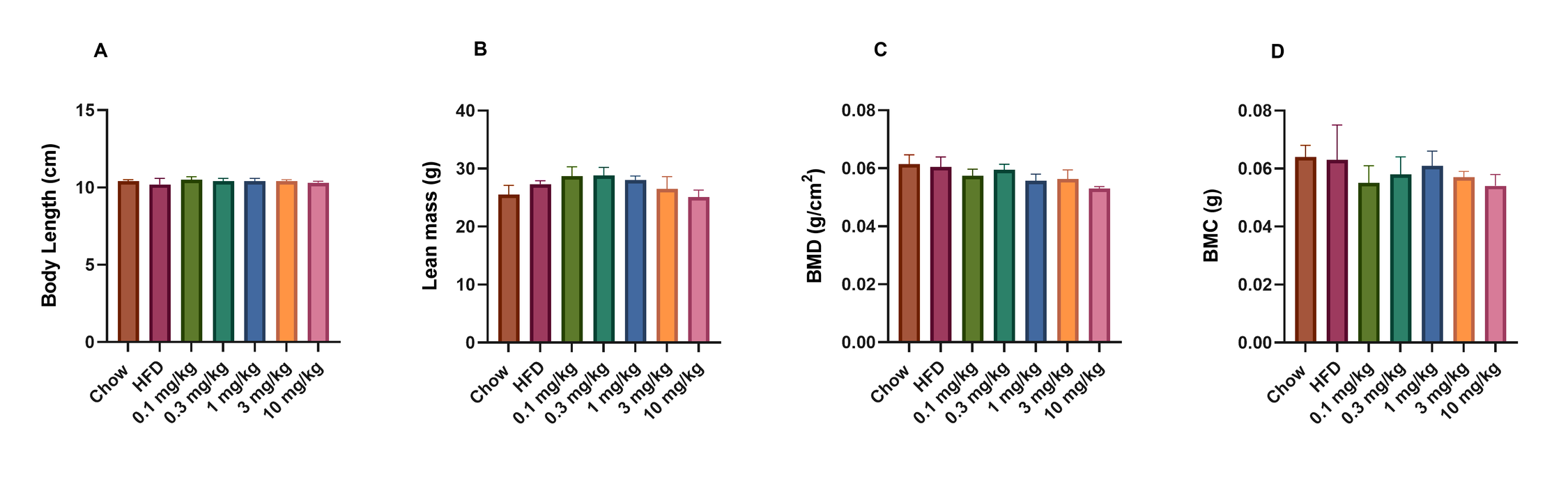

Supplement: S9 Fig — A is the value of the length of the mouse body; B is the lean mass of mice; C is the bone mineral density of mice; D is the bone mineral content of mice. The data are shown as the means ± SDs; n = 5; P < 0.05, **P < 0.01, ***P < 0.001, and ****P < 0.0001 vs the high-diet control group according to one-way ANOVA with Dunnett’s post hoc test. (TIF) [file pone.0331768.s009.tif]

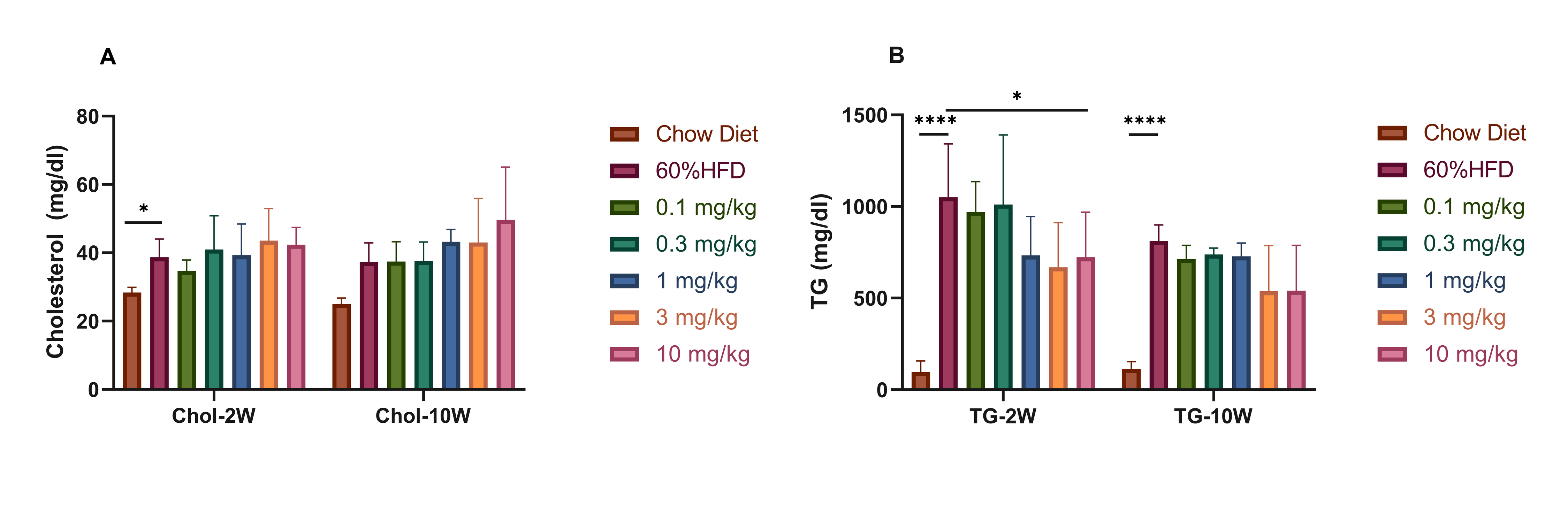

Supplement: S10 Fig — A is the Chol content in liver homogenates of the mice in each group after 2 and 10 weeks of drug intervention; Bis the TG content in liver homogenates of mice in each group after 2 and 10 weeks of drug intervention. The data are shown as the means ± SDs; n = 5; P < 0.05, **P < 0.01, ***P < 0.001, and ****P < 0.0001 vs the high-diet control group according to one-way ANOVA with Dunnett’s post hoc test. (TIF) [file pone.0331768.s010.tif]

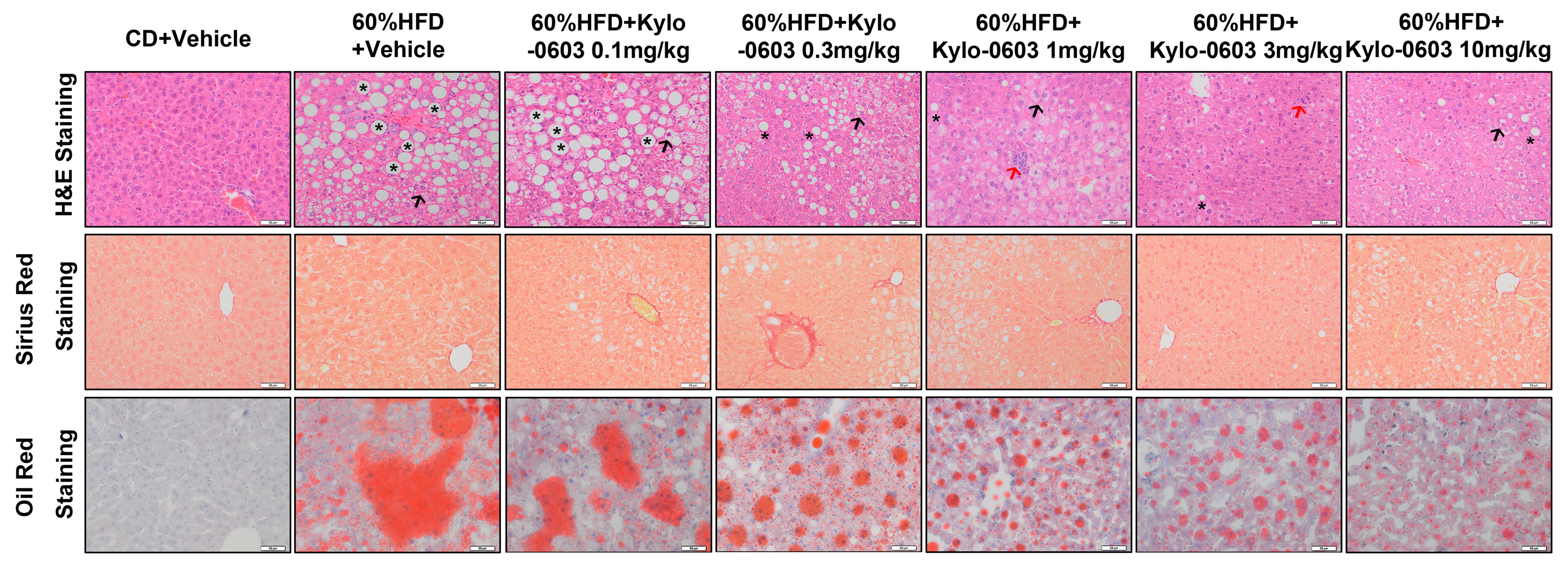

Supplement: S11 Fig — H&E staining, Sirius red staining, and oil red staining of the liver in a diet-induced obese (DIO) mouse model in each group after 8 weeks of pharmacological intervention; steatosis (*), inflammatory foci (red arrows), fibrosis (yellow triangles) and ballooning (blue arrows). Scale bar = 50 μm. n = 8. (TIF) [file pone.0331768.s011.tif]

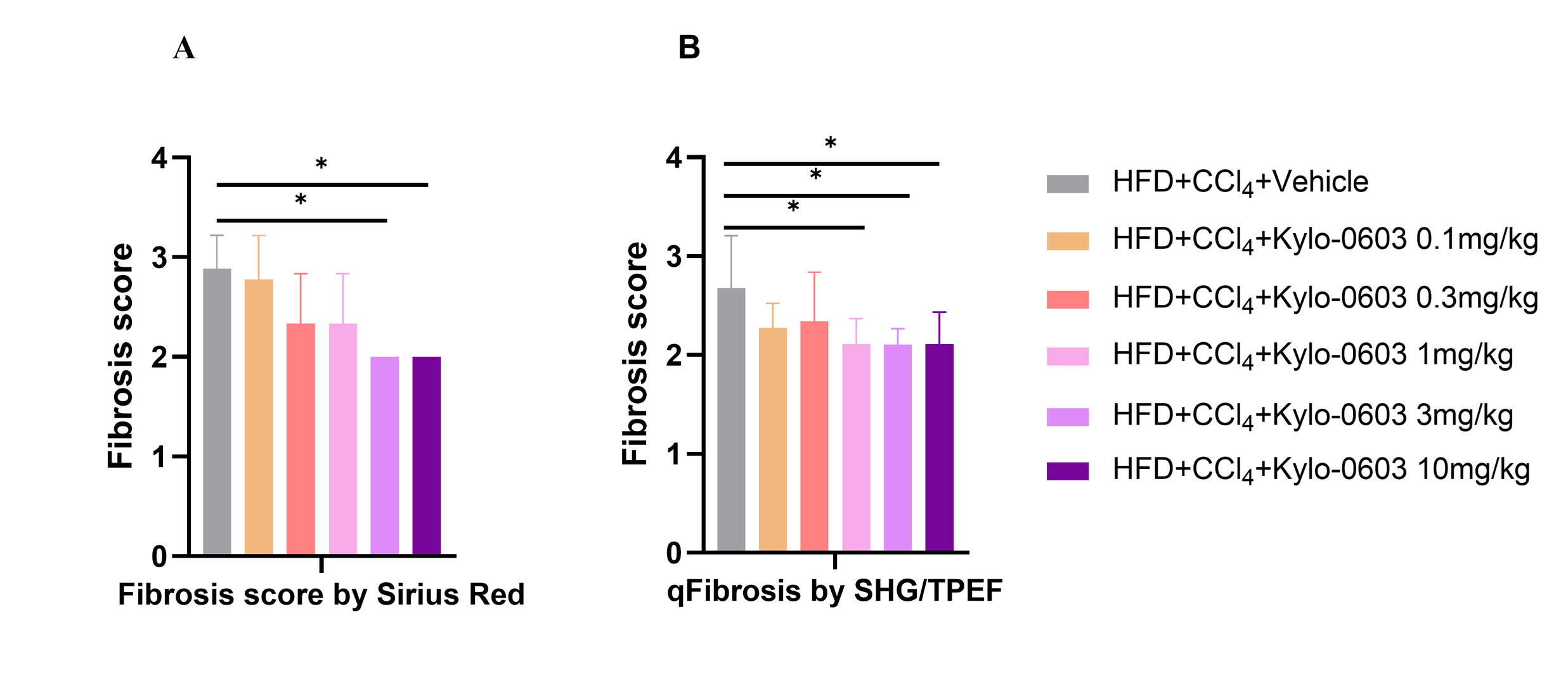

Supplement: S12 Fig — A: Conventional Histopathological Assessment: Fibrosis staging across experimental groups is demonstrated via Sirius Red staining in MASH mouse models (F2 ~ 3). B: SHG/TPEF-Based Fibrosis Quantification: Fibrosis scoring is performed using second harmonic generation/two-photon excitation fluorescence (SHG/TPEF) imaging in MASH mouse models. (TIF) [file pone.0331768.s012.tif]

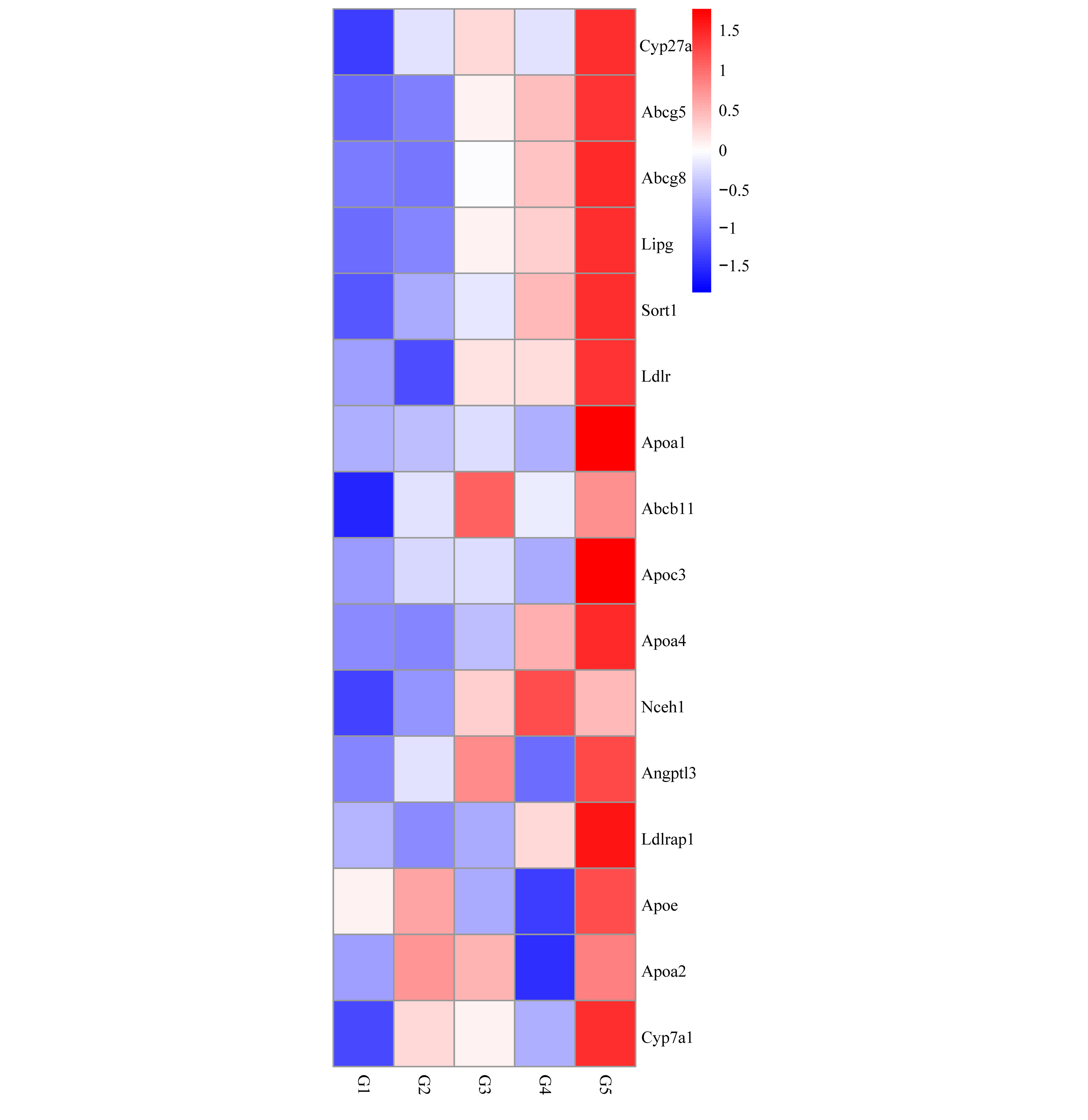

Supplement: S13 Fig — Following a 10-week administration of Kylo-0603 to the HFD + CCl4-induced MASH mouse model, a comparison was conducted between the HFD control group and observed trends in gene expression in liver and cardiac tissue. These trends were related to energy metabolism, lipid accumulation, and hepatic histopathology (covering inflammation and fibrosis) and cardiac function. The aforementioned trends are illustrated in the heatmap displayed below. The figure depicts the dosing groups represented by each symbol, which are as follows: G1 (HFD control), G2 (0.1 mg/kg Kylo-0603), G3 (0.3 mg/kg Kylo-0603), G4 (1 mg/kg Kylo-0603), and G5 (3 mg/kg Kylo-0603). The heatmap illustrates the expression of genes associated with liver tissue, with blue representing low expression and red representing high expression. (TIF) [file pone.0331768.s013.tif]

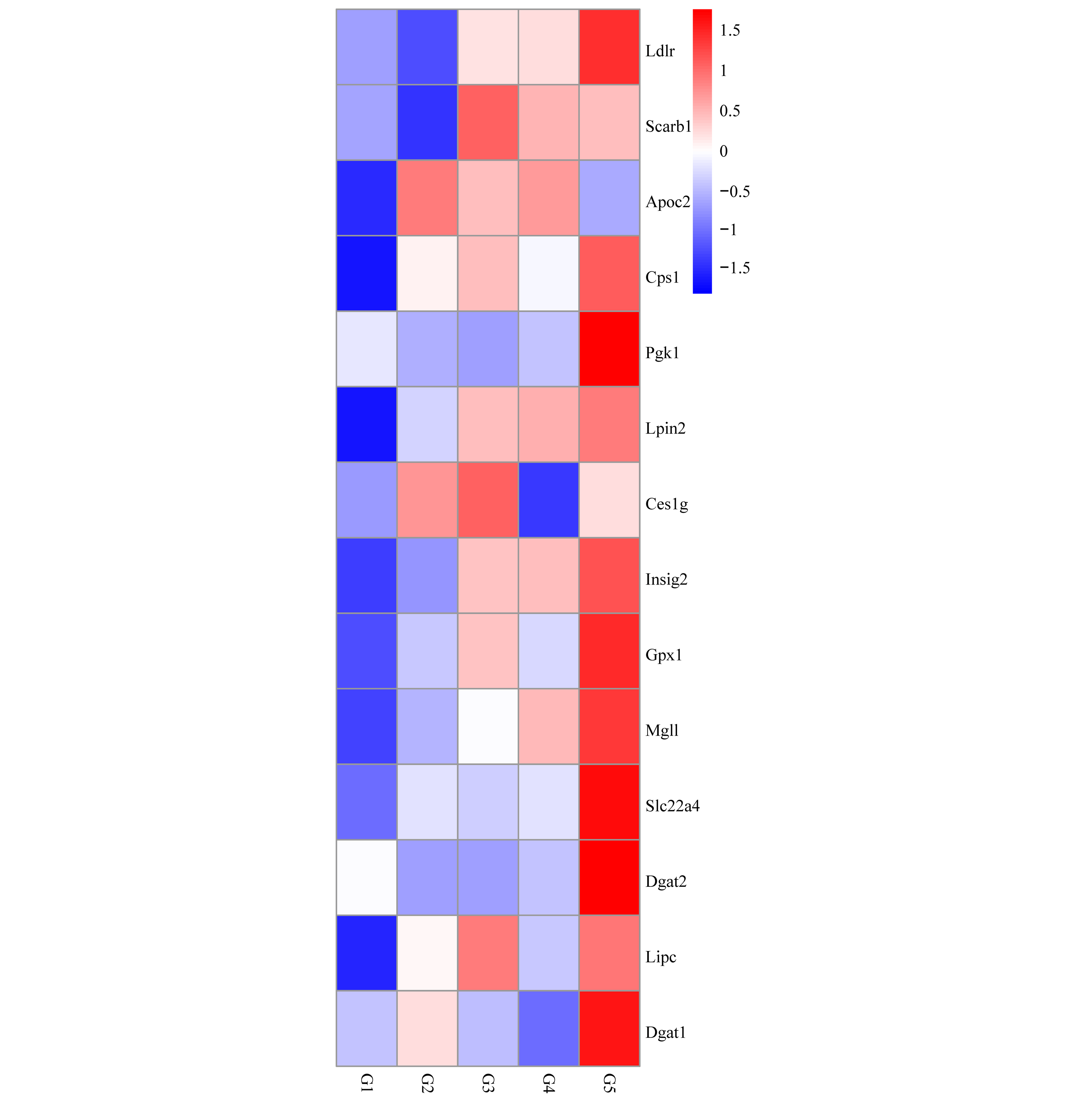

Supplement: S14 Fig — (TIF) [file pone.0331768.s014.tif]

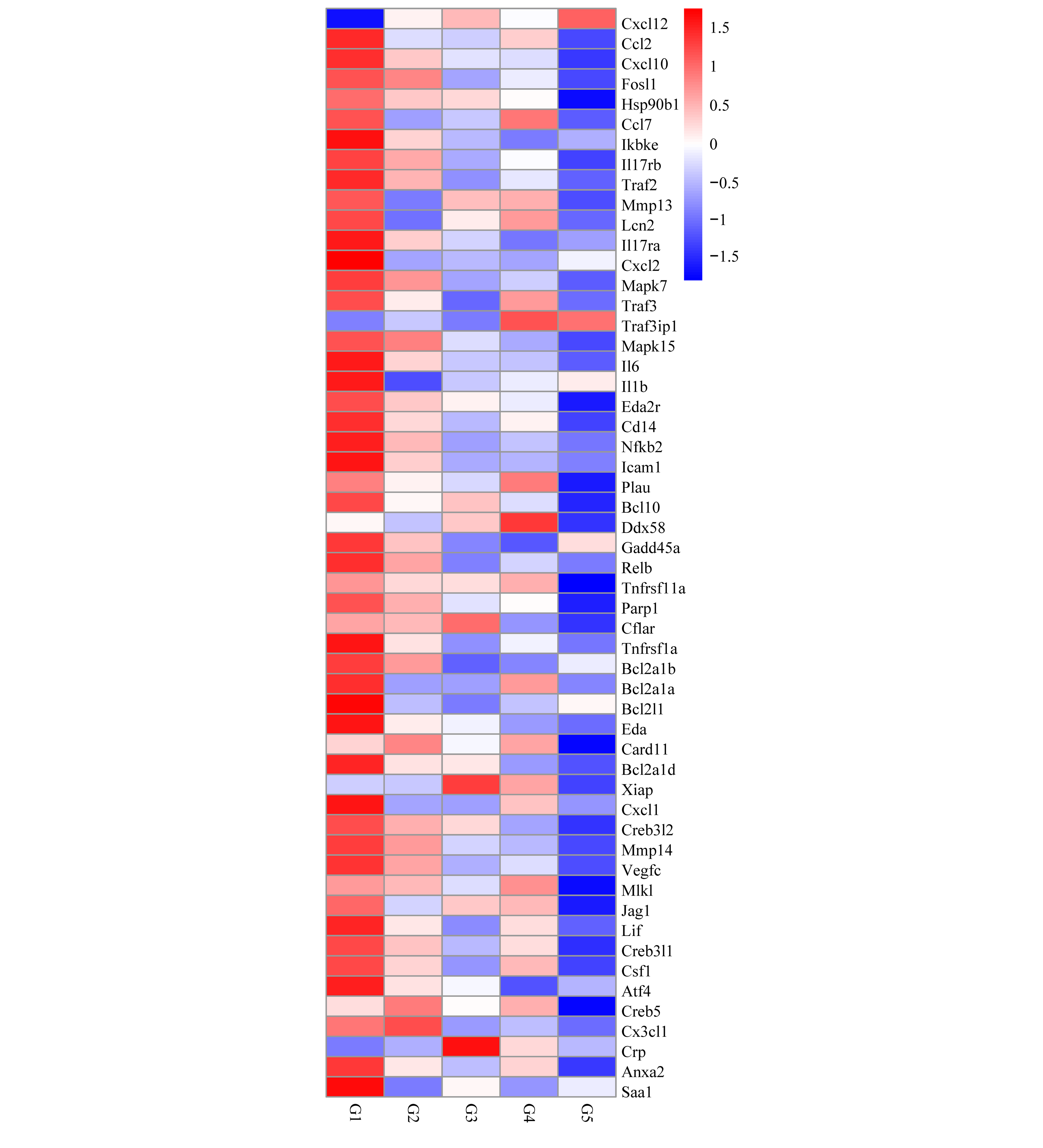

Supplement: S15 Fig — (TIF) [file pone.0331768.s015.tif]

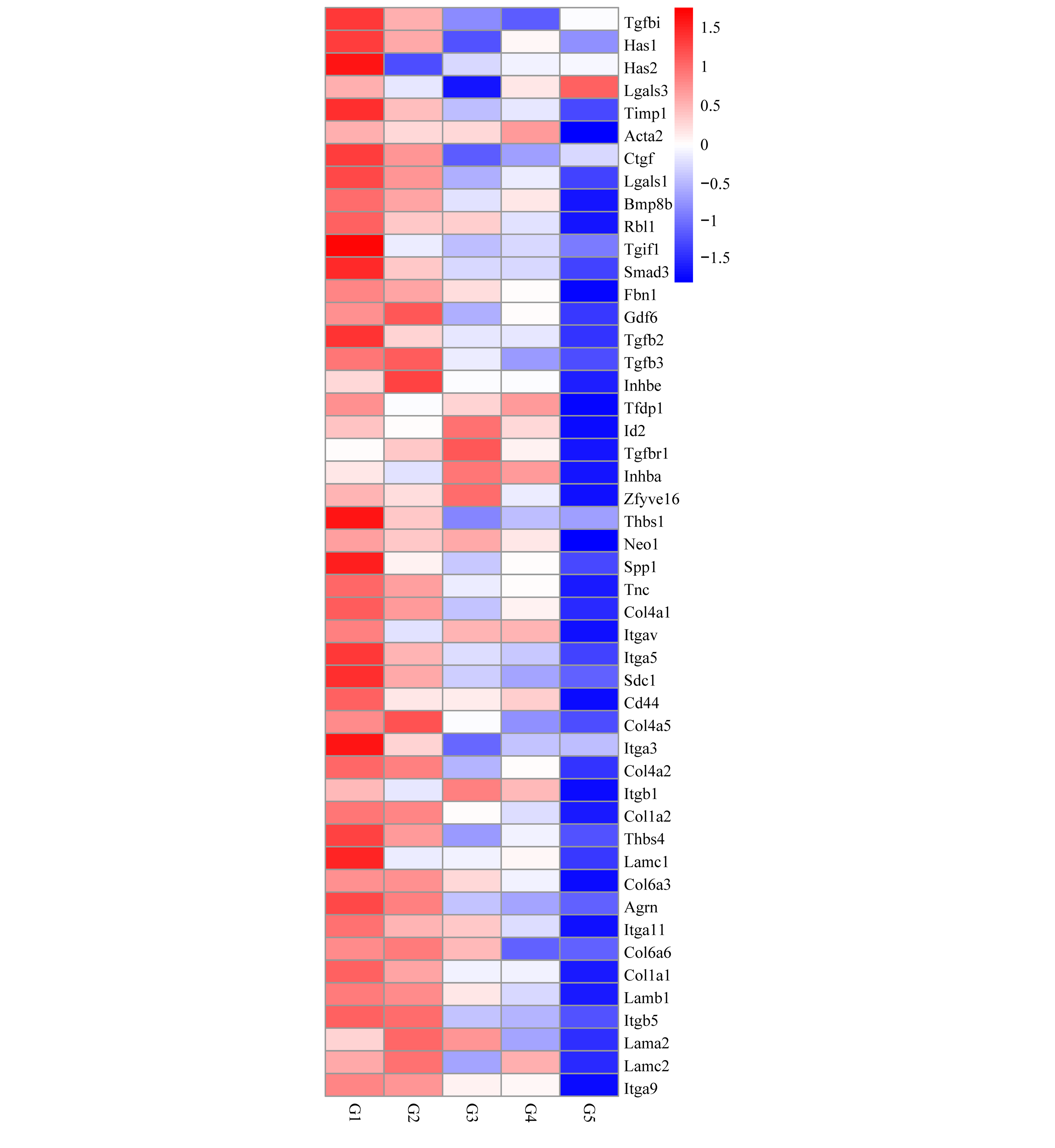

Supplement: S16 Fig — (TIF) [file pone.0331768.s016.tif]

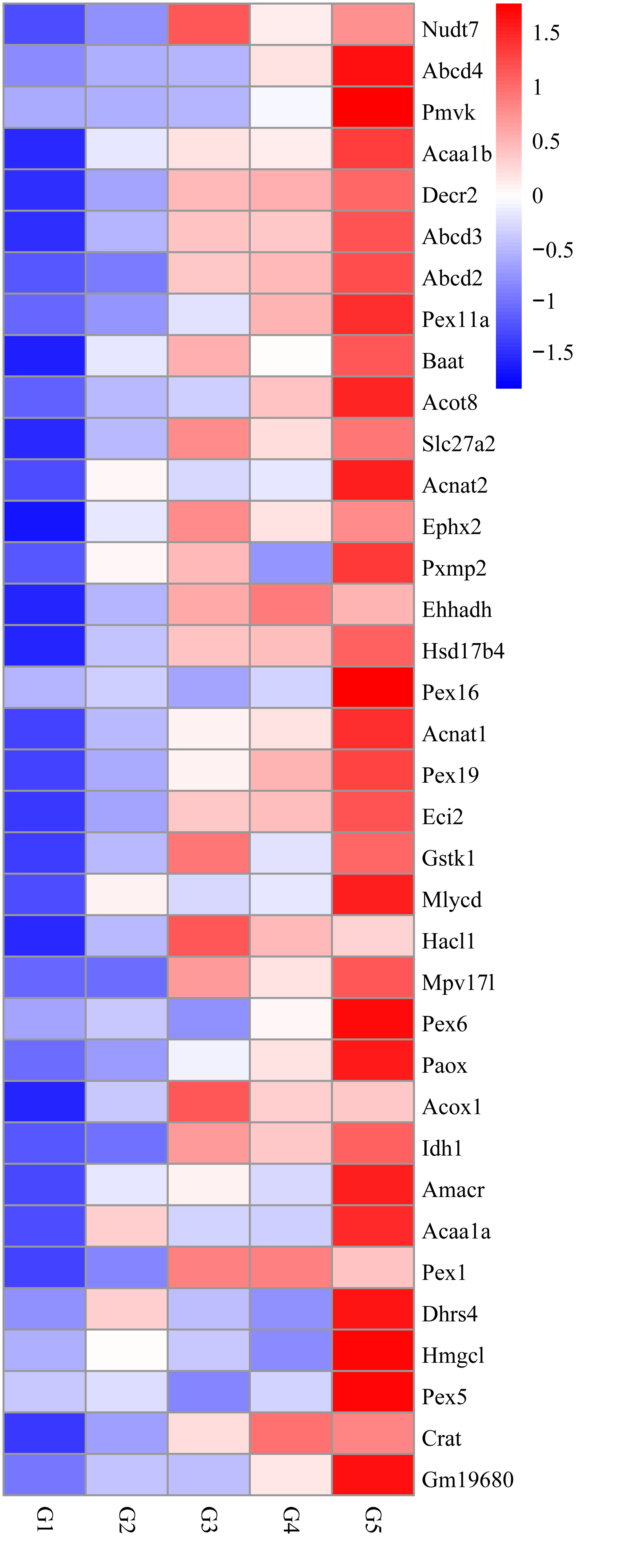

Supplement: S17 Fig — (TIF) [file pone.0331768.s017.tif]
