# Supplementary material for: KYLO-0603, a novel liver-targeting, thyroid hormone receptor-β agonist for the inhibition of MASH progression
Source: PLoS One. 2025 Sep 15;20(9):e0331768. doi: 10.1371/journal.pone.0331768 (PMC12435690; doi:10.1371/journal.pone.0331768)
Supplement: S1 Table — (DOCX) [file pone.0331768.s018.docx]

| Group Number | Number of Animals | Treatment Group | Drug Information | Dose | Administration Route | Administration Frequency |
| --- | --- | --- | --- | --- | --- | --- |
| 1 | 16 | Chow Diet | Vehicle | —— | PO | once daily |
| 2 | 16 | 60% HFD | Vehicle | —— | PO | once daily |
| 3 | 16 | 60% HFD | Kylo-0603 | 0.1mg/kg | PO | once daily |
| 4 | 16 | 60% HFD | Kylo-0603 | 0.3mg/kg | PO | once daily |
| 5 | 16 | 60% HFD | Kylo-0603 | 1mg/kg | PO | once daily |
| 6 | 16 | 60% HFD | Kylo-0603 | 3mg/kg | PO | once daily |
| 7 | 16 | 60% HFD | Kylo-0603 | 10mg/kg | PO | once daily |
