# Supplementary material for: KYLO-0603, a novel liver-targeting, thyroid hormone receptor-β agonist for the inhibition of MASH progression
Source: PLoS One. 2025 Sep 15;20(9):e0331768. doi: 10.1371/journal.pone.0331768 (PMC12435690; doi:10.1371/journal.pone.0331768)
Supplement: S2 Table — Administration volume: 10 μL per gram of body weight (10 μL/g × body weight in grams). (DOCX) [file pone.0331768.s019.docx]

| Group Number | Drug | Dosing (mg/kg) | Concentration (mg/mL) | Preparation Frequency | Drug Preparation |
| --- | --- | --- | --- | --- | --- |
| G1 | Kylo-0603 | 0.1mg/kg | 0.01mg/ml | once every three days | Pipette 0.24 ml of working solution of G5 group, add 23.76 ml of Vehicle, vortex slowly and mix well to get G1 working solution. |
| G2 | Kylo-0603 | 0.3mg/kg | 0.03mg/ml | once every three days | Pipette 0.72 ml of working solution of G5 group, add 23.28 ml of Vehicle, vortex slowly and mix well to get G2 working solution |
| G3 | Kylo-0603 | 1mg/kg | 0.1mg/ml | once every three days | Pipette 2.4 ml of working solution of G5 group, add 21.6 ml of Vehicle, vortex slowly and mix well to get G13 working solution |
| G4 | Kylo-0603 | 3mg/kg | 0.3mg/ml | once every three days | Pipette 7.21 ml of working solution of G5 group, add 16.79 ml of Vehicle, vortex slowly and mix well to get G4 working solution |
| G5 | Kylo-0603 | 10mg/kg | 1mg/ml | once every three days | Weigh 35 mg of Kylo-0603 compound, add 35 ml of vehicle, slowly vortex and mix (to avoid air bubbles) until no particles are present to give a G5 working solution |

**Administration volume:** 10 μL per gram of body weight (10 μL/g × body weight in grams).
