# Supplementary material for: KYLO-0603, a novel liver-targeting, thyroid hormone receptor-β agonist for the inhibition of MASH progression
Source: PLoS One. 2025 Sep 15;20(9):e0331768. doi: 10.1371/journal.pone.0331768 (PMC12435690; doi:10.1371/journal.pone.0331768)
Supplement: S3 Table — (DOCX) [file pone.0331768.s020.docx]

| Group | CD+  Vehicle | HFD+Vehicle | HFD+Kylo-0603 0.1 mg/kg | HFD+Kylo-0603 0.3 mg/kg | HFD+Kylo-0603 1 mg/kg | HFD+Kylo-0603 3 mg/kg | HFD +Kylo-0603 10 mg/kg |
| --- | --- | --- | --- | --- | --- | --- | --- |
| Steatosis | 0.0 ± 0.0*** | 2.3 ± 0.7 | 2.4 ± 1.1 | 2.0 ± 1.4 | 1.5 ± 0.8 | 1.1 ± 1.0 | 1.2 ± 0.8 |
| Lobular inflammation | 0.8 ± 0.5 | 1.0 ± 0.0 | 1.0 ± 0.0 | 1.3 ± 0.5 | 1.1 ± 0.4 | 1.1 ± 0.4 | 1.0 ± 0.0 |
| Ballooning | 0.0 ± 0.0**** | 1.5 ± 0.5 | 1.6 ± 0.5 | 1.4 ± 0.5 | 1.8 ± 0.5 | 1.1 ± 1.0 | 1.0 ± 0.9 |
| NAFLD activity score | 0.8 ± 0.5**** | 4.8 ± 0.9 | 5.0 ± 0.9 | 4.6 ± 1.2 | 4.4 ± 1.1 | 3.4 ± 2.1 | 3.2 ± 1.3 |
| Fibrosis Score | 0.1 ± 0.4 | 0.4 ± 0.7 | 0.6 ± 0.5 | 0.4 ± 0.5 | 0.4 ± 0.5 | 0.4 ± 0.5 | 0.3 ± 0.5 |
