# Supplementary material for: KYLO-0603, a novel liver-targeting, thyroid hormone receptor-β agonist for the inhibition of MASH progression
Source: PLoS One. 2025 Sep 15;20(9):e0331768. doi: 10.1371/journal.pone.0331768 (PMC12435690; doi:10.1371/journal.pone.0331768)
Supplement: S4 Table — The detailed experimental design and results are as follows: After a single oral administration of 12.5 mg/200 μCi/kg [14C] Kylo-0603 to SD rats (n = 6, 3 males and 3 females per group), the compound in tissues were detected by semi-quantitive radioactivities from 0 to 168 hours. The experiment results showed that 94.2% of the radioactivity was excreted via feces, with only 0.05% excreted in urine. For the tissue distribution study, the same dosing regimen was used to measure the amount of radioactivity in various tissues at 0.083, 0.25, 1, 4 and 24 hours (n = 6; three females and three males at each time point). The data showed that the highest concentrations of radioactivity were found in the liver, followed by the stomach, small intestine and large intestine. Very low levels were found in the spleen, kidney, lung and adipose tissue. Concentrations in bone, the thyroid gland, the thymus gland, the heart, skeletal muscle and the whole brain were all below the limit of detection. These data collectively confirm the significant liver-targeting property of Kylo-0603. (DOCX) [file pone.0331768.s021.docx]

| Total Radioactivity (ngEq/g) | | | | | | |
| --- | --- | --- | --- | --- | --- | --- |
| Tissue | 0.083 h | 0.25h | 1h | 4h | 8h | 24h |
| Skeletal Muscle | BLQ | BLQ | BLQ | BLQ | BLQ | BLQ |
| Fat | BLQ | 3.49±4.7 | 2.41±2.3 | BLQ | BLQ | BLQ |
| Thyroid Gland | BLQ | BLQ | BLQ | BLQ | BLQ | BLQ |
| Whole Brain | BLQ | BLQ | BLQ | BLQ | BLQ | BLQ |
| Thymus | BLQ | BLQ | BLQ | BLQ | BLQ | BLQ |
| Heart | BLQ | BLQ | BLQ | BLQ | BLQ | BLQ |
| Spleen | BLQ | 20.5±3.07 | 41.2±1.89 | BLQ | BLQ | BLQ |
| Lung | 23.91±3.5 | BLQ | BLQ | BLQ | BLQ | BLQ |
| Kidney | BLQ | BLQ | 8.37±69.3 | BLQ | BLQ | BLQ |
| Liver | BLQ | 141±50.5 | 413±24.8 | BLQ | BLQ | BLQ |

BLQ：below the lower limit of quantification
